# Stroke by inducing HDAC9-dependent deacetylation of HIF-1 and Sp1, promotes TfR1 transcription and GPX4 reduction, thus determining ferroptotic neuronal death

**DOI:** 10.7150/ijbs.80735

**Published:** 2023-05-11

**Authors:** Luca Sanguigno, Natascia Guida, Serenella Anzilotti, Ornella Cuomo, Luigi Mascolo, Angelo Serani, Paola Brancaccio, Giuseppina Pennacchio, Ester Licastro, Giuseppe Pignataro, Pasquale Molinaro, Lucio Annunziato, Luigi Formisano

**Affiliations:** 1 Division of Pharmacology, Department of Neuroscience, Reproductive and Dentistry Sciences, School of Medicine, Federico II University of Naples, Via Pansini, 5, 80131, Naples, Italy.; 2 Division of Pharmacology, Department of Science and Technology, University of Sannio, 82100 Benevento, Italy.; 3 IRCCS Synlab SDN S.p.A, Via Gianturco 113, 80143 Naples, Italy.

**Keywords:** HDAC9, Ferroptosis, Stroke, GPX4, TfR1

## Abstract

**Background:** The inhibition of histone deacetylase 9 (HDAC9) represents a promising druggable target for stroke intervention. Indeed, HDAC9 is overexpressed in neurons after brain ischemia where exerts a neurodetrimental role. However, mechanisms of HDAC9-dependent neuronal cell death are not yet well established.

**Methods:** Brain ischemia was obtained *in vitro* by primary cortical neurons exposed to glucose deprivation plus reoxygenation (OGD/Rx) and *in vivo* by transient middle cerebral artery occlusion. Western blot and quantitative real-time polymerase chain reaction were used to evaluate transcript and protein levels. Chromatin immunoprecipitation was used to evaluate the binding of transcription factors to the promoter of target genes. Cell viability was measured by MTT and LDH assays. Ferroptosis was evaluated by iron overload and 4-hydroxynonenal (4-HNE) release.

**Results:** Our results showed that HDAC9 binds to hypoxia-inducible factor 1 (HIF-1) and specificity protein 1 (Sp1), two transcription activators of transferrin 1 receptor (TfR1) and glutathione peroxidase 4 (GPX4) genes, respectively, in neuronal cells exposed to OGD/Rx. Consequently, HDAC9 induced: (1) an increase in protein level of HIF-1 by deacetylation and deubiquitination, thus promoting the transcription of the pro-ferroptotic TfR1 gene; and (2) a reduction in Sp1 protein levels by deacetylation and ubiquitination, thus resulting in a down-regulation of the anti-ferroptotic GPX4 gene. Supporting these results, the silencing of HDAC9 partially prevented either HIF-1 increase and Sp1 reduction after OGD/Rx. Interestingly, silencing of the neurodetrimental factors, HDAC9, HIF-1, or TfR1 or the overexpression of the prosurvival factors Sp1 or GPX4 significantly reduced a well-known marker of ferroptosis 4-HNE after OGD/Rx.

More important, *in vivo*, intracerebroventricular injection of siHDAC9 reduced 4-HNE levels after stroke by preventing: (1) HIF-1 and TfR1 increase and thus the augmented intracellular iron overload; and (2) a reduction of Sp1 and its target gene GPX4.

**Conclusions:** Collectively, results obtained suggest that HDAC9 mediates post-traslational modifications of HIF-1 and Sp1 that, in turn, increases TfR1 and decreases GPX4 expression, thus promoting neuronal ferroptosis in *in vitro* and *in vivo* models of stroke.

## Introduction

Histone deacetylases (HDACs) are enzymes regulating the process of transcription by catalyzing the removal of acetyl functional groups from the lysine residues of both histone and non-histone proteins such as transcription factors 1. They are divided in four different classes: class I HDACs (1-3 and 8) are constitutively expressed in nucleus; class II HDACs (4-7, 9, and 10) shuttle between the nucleus and cytoplasm; class III HDACs, also known as sirtuins, consist of seven members (SIRT1-SIRT7); and class IV HDAC, that is currently composed of one member, HDAC11 2. Among the several isoforms of HDACs, HDAC9 has been found to play a relevant role in the neuronal death mechanisms which are activated in stroke. HDAC9 is expressed in a variety of cell types involved in stroke pathophysiology including glial, endothelial, smooth muscle and neuronal cells 3. Interestingly, several studies showed that there is an higher frequency of a single-nucleotide polymorphism in HDAC9 gene, *rs2107595*, in cohorts of patients suffering of: (1) large artery stroke 4, 5; (2) asymptomatic carotid plaque 6 and (3) vascular calcification 7. Interestingly, it has been suggested that *rs2107595* polymorphism is able to increase HDAC9 promoter activity 8). It should be noted that HDAC9 can be induced, not only by *rs2107595* polymorphism, but also by some pathophysiological conditions, such as human carotid atherosclerotic plaques 9 and stroke 10-12.

At neuronal level, HDAC9 is expressed specifically in post-mitotic neurons, but not in adult neuronal stem cells 13. Interestingly, HDAC9 is up-regulated during stroke where it participates in increasing the final infarct volume size by: (1) suppressing autophagy 12, (2) activating IκBα/NF-κB and MAPKs signaling pathway 11 and (3) reducing miR-20a expression with a consequent up-regulation of its target gene Neurod1 10. Furthermore, the HDAC class IIa inhibitor TMP269 improves neurological score and infarct volume after stroke by reducing HDAC9 protein expression 14. In addition, the inhibition of HDACs reduces neuronal cell death after glutamate excitotoxicity 15 by counteracting the activation of the ferroptotic pathway 16. In particular, ferroptosis is a type of cell death characterized by iron-dependent accumulation of lipid reactive oxygen species (L-ROS). It is well-known that the intracellular iron concentrations are regulated by three proteins: (1) the iron uptake protein transferrin receptor 1 (TfR1), that is responsible for the amount of iron uptake; (2) the iron release protein ferroportin 1 (Fpn1), that determines the amount of released iron; (3) the iron storage protein ferritin (Ft), that participate to the amount of sequestered iron 17-19. Cell death occurs when the intracellular levels of L-ROS exceed the antioxidant activity of glutathione-dependent peroxidase (GPX4), thus leading to the collapse of cellular redox homeostasis. GPX4 is a unique enzyme able to reduce oxidized lipids (L-OOH) to harmless lipid alcohols (L-OH) by converting glutathione (GSH) into oxidized glutathione (GSSG) 20.

Interestingly, the counteraction of either the increase in TfR1 or the reduction in Fpn1, Ft and GPX4, at transcriptional or translational level, causes an amelioration of neuronal damage after transient middle cerebral artery occlusion (tMCAO) 21. Intriguingly, TfR1, Fpn1, Ft are target genes of the transcription factor HIF-1 22-24, whereas GPX4 is regulated by the transcription activator Sp1. HIF-1 potentiates iron uptake and overload by enhancing the expression of TfR1, and thus promoting neuronal death after stroke 25. On the other hand, the increase in Sp1 binding on GPX4 promoter leads to protection from oxidative stress in *in vitro* and *in vivo* models of thrombotic and hemorrhagic stroke 26.

In the light of these premises and taking into account that: (1) HDAC9 is neurodetrimental in stroke 27; (2) HDACs, including HDAC9, can deacetylate transcription factors on lysine residues 28; (3) TfR1, Fpn1, and Ft expression are up-regulated by HIF-1; and (4) GPX4 transcription is up-regulated by Sp1; the aim of the present work was to determine the role played by HIF-1, Sp1, TfR1 and GPX4 in the signaling of ferroptotic neuronal death mediated by HDAC9 in *in vitro* and *in vivo* models of stroke.

## Material and Methods

### Reagents and cell cultures

All reagents were obtained from Sigma (Milan, IT). DNA primers were synthesized by Eurofins Genomics (Ebersberg, Germany). The following siRNAs have been used: (1) human HDAC9 (EHU090071), (2) mouse HDAC9 (EMU19511), but also (3) rat siRNAs for HDAC9, SUMO1, HDAC4 and HDAC5, 29, HIF-1, HDAC1, HDAC2 30 and TfR1 31, as previously reported. For HDAC9 plasmid overexpression, the construct was kindly provided by Prof. Edward Seto (Washington University, USA) 32, whereas GPX4 vector (cod: HG18231-CF) from Sinobiological (Milan, IT). Sp1 vector for overexpression was gently gift from Prof. Suske from Institute of Molecular Biology and Tumor Research (IMT), Marburg, Germany 33. The plasmid pcDNA3.1 was used as empty vector (mock). Human neuronal-like SH-SY5Y, human glial-like U87 and mouse endothelial-like bEND3 cells were purchased from (IRCCS Azienda Ospedaliera Universitaria San Martino-IST Istituto Nazionale per la Ricerca sul Cancro, Genova, Italy). SH-SY5Y and U87 cells were grown as previously published 32, whereas bEND3 cells was cultured in DMEM / 10% FBS/1 × Pen-Strep at 37 °C in a humidified 5% CO_2_ incubator. The medium of culture was changed every two days. All experiments for SH-SY5Y, U87 and bEND3 were performed on cells at passages 8 to 20.

Primary rat cortical neurons were prepared as previously reported and used at 7-9 days in vitro (DIV) 32. After 48 hours of seeding, neurons were exposed to cytosine arabinoside (2.5 µM) to reduce glial contamination. Experiments on primary cortical neurons were performed according to the procedures described in experimental protocols approved by Ethical Committee of the “Federico II” University of Naples. Both MC1568 (M1824) and MG132 (M8699) were purchased from Sigma and diluted in cell culture medium, the final DMSO concentration was 1%. DMSO was added to the control cells (vehicle) at the same concentration as that used for treated cells. The addition of DMSO alone caused no cellular toxicity.

The density of SH-SY5Y, U87 and cortical neuronal cells used in the experiments were already previously described 32, while for bEND3 was 2x10^6^ cells/well for 12-well plate for MTT and LDH assays and 5x10^6^ cells/well for 60 mm plate for qRT-PCR and 12x10^6^ cells/well for 100 mm plate for ChIP and Western Blot analysis.

### Transfection with small interfering RNA or plasmids in cell cultures

Human, rat and mouse siRNAs for HDAC9, TfR1, HIF-1 and siCTL were transfected at the concentration of 50 nM. Each transfection was achieved in Opti-MEM plus Lipofectamine LTX (15338100, Thermo Fisher), as recommended by the producer. Cortical neurons were transfected at 5 days in vitro (DIV), while SH-SY5Y, U87 and bEND3 cells were transfected 24 hours after plating. For HDAC9, Sp1 and GPX4 overexpression experiments, cortical neurons were transfected with 15 μg for 100mm plates, 5 μg for 60mm plates and 1 μg for mw12 plates of DNA or the empty vector pcDNA3 (mock) using Lipofectamine LTX in Optimem, 24 h before OGD/Rx. Transfection efficiency of siRNAs or constructs was evaluated by qRT-PCR 34 to assess the levels of human GPX4, mouse HDAC9, rat HDAC9 mRNAs, whereas the effect of rat siHIF-1 and human siHDAC9 transfection to reduce the HIF-1 and HDAC9 transcripts levels and of Sp1 vector to increase its mRNA levels were already published 29, 30

### Combined oxygen and glucose deprivation (OGD) and reoxygenation (Rx) and determination of cell survival

OGD/Rx was performed in SH-SY5Y, U87, bEND3 cells and rat cortical neurons as previously reported 35, 36. Cells were incubated in a medium containing: 116 mM NaCl, 5.4 mM KCl, 0.8 mM MgSO4, 26.2 mM NaHCO3, 1 mM NaH2PO4, 1.8 mM CaCl2, 0.01 mM glycine, and 0.001% w/v phenol red, this medium is saturated with 95% N2 and 5% CO2 at 37°C for 20 minutes before adding it to the cells. Afterward, SH- SY5Y, U87, bEND3 and cortical neurons were placed in a hypoxic chamber for 4 hours, in the following condition: temperature 37°C, atmosphere 5% CO2, and 95% N2. To block OGD, cells were removed from the hypoxic chamber and placed in a normal medium for 24 and 48 hours of reoxygenation (Rx) for cortical neurons and for 48 and 72 hours of Rx for SH-SY5Y, U87, and bEND3 cells. Transfections with siRNAs for SH-SY5Y, U87, bEND3 cells were performed 24 hours after plating; OGD/Rx procedure starts 24 hours after transfection. For the experiments with MG132, cells were pre-treated with the proteasome inhibitor MG132 (10 μM) for two hours before OGD/Rx exposure. Transfections with siRNAs or constructs for cortical neurons were performed at 5 DIV; cells were subjected to OGD/Rx 24 hours after transfection. MC1568 (5 μM) was added 1 hour before and during the entire OGD phase. Neuronal survival was revealed as previously described, by using the MTT staining and LDH assay 32. For MTT assay the medium was eliminated and cells were incubated in 0.5 mg/ml MTT solution for 2 h at 37 °C. The incubation was blocked by adding 500 μl of isopropanol to solubilize the formazan salt, and viability was read by measuring the absorbance at 540 nm with a spectrophotometer. LDH efflux was measured into the cellular medium in SH-SY5Y, U87, and bEND3 with the LDH cytotoxicity Kitf rom Cayman, DBA (Milan, Italy) as previously reported 32. Cells treated with 1% Triton X-100 (Sigma-Aldrich) was used as a positive control and its value was considered 100% cellular death.

### 4-HNE assay

For the evaluation of oxidative stress, frozen cortex or cell samples were lysed with cold PBS with the use of syringes and resuspended in a volume of PBS equal to nine times the weight and kept on ice. After, the samples were centrifuged at 10.000 g for 10 minutes and the supernatants were recovered. Then the samples are used for the 4-HNE evaluation according to the manufacturer's instructions (E-EL-0128 - Elabscience). Data were obtained on the basis of a standards curve.

### Total iron assay

For the evaluation of total iron, frozen cortex tissues were homogenized with cold reagent 1 (buffer solution of colorimetric assay kit) with the use of syringes. Tissue samples were homogenized with a volume of reagent 1 equal to nine times the weight, and kept on ice. After, lysates were centrifuged at 10.000 g for 10 minutes and the supernatants were recovered. Then the samples are used for the total iron colorimetric assay according to the manufacturer's instructions (E-BC-K772-M - Elabscience). Data were obtained on the basis of a standards curve.

### Quantitative Real-Time PCR (qPCR) Analysis

qPCR experiments were executed as previously reported 37. After RNA retrotranscription, the obtained DNA at the same concentration was amplified concurrently in duplicate in one assay as follows: heating 2 min @ 50º C, denaturation 10 min @ 95º C, amplification and quantification 35 cycles of 15 sec @ 95º C, 30sec @ 60º C with a single fluorescence measurement. PCR data was collected using ABI Prism 7000 SDS software (Applied Biosystems). Normalization of the data was performed by Ribosomal Protein L19 for human genes and hypoxanthine-phosphoribosyltransferase (HPRT) for mouse and rat genes as an internal control 32. The primers used for this work were the following: HDAC9 (H) (FW 5'-GAATTAGAATGCGTTGCTGTGAA-3' e RV 5'-CCAAACAATGGGCCAACTG-3'); Fpn1 (H) (FW 5′-TCTCGCTGAGGTGCTTGTTAAC-3′ and RV 5′-GGCTGTTGTGTTTTT-3′); GPX4 (H) (FW 5′-CGCCGCGGGCTAACA-3′ and RV 5′-AACCATGTGCCCGTCGAT-3′); TFR1 (H) (FW 5′-CCATTGTCATATACCCGGTTCA-3′ and RV 5′-CCACATGACTGTTATCGCCATCT-3′); Ferritin heavy chain (Ft) (H) (FW 5′-GGTGGCCGAATCTTCCTTCA-3′ and RV 5′-GCCAGTTTGTGCAGTTCCAG-3′); HDAC9 (M) (FW 5′-ATTGACATCCAAAGCAAGTATTGG-3′ and RV 5′-GCACCGCCCCTTGCA-3′); Fpn1 (M) (FW 5′-TCTCGCTGAGGTGCTTGTTAAC-3′ and RV 5′-GGGTGGATAAGAATGCCAGACT-3′); GPX4 (M) (FW 5′-GCTGGGCTAGTCCTGAGAATAGAC-3′ and RV 5′-GCTGTGCGCGCTCCAT-3′); TfR1 (M) (FW 5′-CTCAGTTTCCGCCATCTCAGT-3′ and RV 5′-GCAGCTCTTGAGATTGTTTGCA-3′); Ferritin heavy chain (Ft) (M) (FW 5′-CTGGAACTGCACAAACTGGC-3′ and RV 5′-CTCTCATCACCGTGTCCCAG-3′); HDAC9 (R) (FW 5′-CCAGGGTCAGGTCCCAGTT-3′ and RV 5′-CCTCGTTTTCGGTAACATTTCC-3′); GPX4 (R) (FW 5′-GCTGTGCGCGCTCCAT-3′ and RV 5′-ACCATGTGCCCATCGATGT-3′); TfR1 (R) (FW 5′-GAATACGTTCCCCGTTGTTGA-3′ and RV 5′-ATCCCCAGTTCCTAGATGAGCAT-3′); HIF-1 (R) (FW 5′-CGAGCTGCCTCTTCGACAAG-3′ and RV 5′-CGCTGGAGCTAGCAGAGTCA-3′). Modifications in mRNA levels among groups were showed as the mean of the relative quantification (RQ) values, that was obtained as the difference in threshold cycle (ΔCt) between the target gene and the reference gene (2^-ΔΔCt^ =RQ).

### Chromatin immunoprecipitation (ChIP) Analysis

Chromatin lysates from cells and brain tissues were incubated overnight with 3µg of antibody for HIF-1 and Sp1 30. Genomic coordinates for HIF-1 binding site on TfR1 gene are: for mouse (Chr16:v32,427,402-32,427,410, GRCm39), for human (Chr3:196.082.481-196.082.488, GRCh38.p13) and rat (Chr11:68.185.655-68.185.663, mRatBN7.2). Genomic coordinates for Sp1 binding site on GPX4 gene are: for mouse (Chr10:79.889.279-79.889.290, GRCm39), for human (Chr19:1.103.926-1.103.915, GRCh38.p13) and rat (Chr7:9.652.996-9.653.007, mRatBN7.2). Chromatin of cells were also incubated with 3µg of HDAC9 antibody (sc-28732; Santa Cruz Biotechnology, CA, USA). Mouse or rabbit IgG were used as negative control. Immunoprecipitated DNA was analyzed by q-PCR using Fast SYBR Green Master Mix (cod 4385610; Applied Biosystems, Italy). The primers used for the amplification of the immunoprecipitated DNA were: human GPX4 promoters (FW (H) 5′-CTGTACAGGGGTCAAAGTCCA-3′ and RV (H) 5′-CCCACATCTTGGCTTCCAACT-3′; mouse GPX4 promoters (FW (M) 5′-CTGCTCTTCCAGAGGTCCTG-3′ and RV (M) 5′-GAGGTGTCCACCAGAGAAGC-3′), whereas for human and mouse TfR1 promoters were: (FW (H) 5′-CTTGGCGCCTTTTCCCTTG-3′ and RV (H) 5′-GTCACTTCCTGAGGCACGTA -3′; FW (M) 5′-CGAGGGGCCCCAAGG-3′ and RV (M) 5′-CGAGAGTCACCACGCTGAG-3′). For mouse Beclin promoter we used primers already used by 38. Specifically, samples were heated for 2 min at 50°C and denatured for 10 min at 95°C. Amplification and quantification, as previously reported 39 and graphically represented as percentage of control or sham.

### Western Blotting and Co-Immunoprecipitation

Experiments for Western Blotting, Co-Immunoprecipitation and Nucleus/Cytosol fractionation were performed as previously reported 32. Proteins for all experiments were separated on 4-15% precast polyacrylamide gel (Biorad). All were transferred onto nitrocellulose membranes using the Trans-Blot® SD Semi-Dry Transfer Cell (Biorad). Membranes were blocked with 5% non-fat dry milk in Tris-buffered saline with Tween 20 (tTBS) for 2 hours at room temperature, and then they were incubated overnight at 4°C with the following antibodies: 1:500 anti-HDAC9 (sc-28732), anti-TfR1 (sc-65882) and anti-HIF-1 (sc-13515) (Santa Cruz Biotechnology, INC), 1:1000 anti-GPX4 (SAB4300725) (Sigma, Milan, IT). Antibodies against HDAC1, HDAC2, HDAC4, HDAC5, Sp1, β-actin were previously published 30, 32.

For Nucleus/Cytosol fractionation two plates each containing 5×10^6^ for SH-SY5Y cells were washed with cold PBS and collected. The cell pellet was resuspended in 500 µl of hypotonic buffer (10 mM Hepes KOH pH 7.9, 10 mM KCl, 1.5 mM MgCl_2_, 0.1 mM EGTA, 0.5 mM DTT, 1 mM NaV_3_O_4_ and 0.2 mM PMSF) and incubated on ice for 15 min. Then cells were centrifuged at 3000 rpm for 5 min and the pellet was resuspended in hypotonic buffer. Next, cells were passed 8-10 times through a 1-ml syringe. The homogenate was centrifuged for 10 min at 1000 rpm at 4 °C to obtain the cytoplasmic fraction (supernatant) and nuclear fraction (pellet). The nuclear pellet was resuspended in 50 µl of complete cell extraction buffer (20 mM Hepes KOH pH 7.9, 0.4 M NaCl, 1.5 mM MgCl_2_, 0.1 mM EGTA, 25% glycerol, 0.5 mM DTT, 1 mM NaV_3_O_4_, 0.2 mM PMSF, 0.5% deoxycholate) and incubated on ice for 30 min with vortexing at 10 min intervals. The nuclear lysate was centrifuged at 14000 rpm for 30 min at 4 °C to obtain the nuclear fraction (supernatant). For immunoprecipitation lysates of SH-SY5Y of 1500μg were immunoprecipitated overnight at 4°C using 3μg anti anti-Sp1 or anti-HIF-1 and normal IgG, as negative control. The immunoprecipitates were then subjected to Western blot analysis as above described. Membranes were incubated overnight at 4°C with anti-HDAC9 (ab-18970) (Abcam, USA), anti-ubiquitin (1:1000, cat: sc-8017, Santa Cruz Biotechnology Inc.), anti-acetyl-lysine (1:1000, cat: 9441, Cell Signaling). For immunoprecipitation lysates of cortical neurons of 1000μg were immunoprecipitated overnight at 4°C with 3μg of anti-SUMO1 37 and normal IgG, as negative control. Membrane was incubated overnight at 4°C with anti-HDAC9 (ab-18970).

### In vivo studies

#### Experimental Groups

Forty-two C57 male 8-week-old mice (Charles River) weighting 22-24 gr were housed under diurnal lighting conditions (12h darkness/light). Mice did not show a reduction in cerebral blood flow of at least 70% and those dying after stroke induction, as well as animals that died after ischemia induction, that signify 20% of total animals used, were eliminated from the study. Sample size for cerebral ischemia experiments measurements was calculated a priori (n=5/group) by predicting a conventional effect size of 0.9 and an alpha error of 0.1 and a statistical power of 95%. Experiments were performed according to the international guidelines for animal research and approved by the Animal Care Committee of Federico II University of Naples, Italy.

#### Focal Transient Brain Ischemia and siRNA administration

Transient focal ischemia was induced as previously described 32, by occlusion of the middle cerebral artery (MCA) in male mice anesthetized using 1.5 % sevoflurane, and 98.5% O2. Specifically, a 5-O surgical monofilament nylon suture (Doccol, CA, USA) was inserted through the external carotid artery stump and advanced into the left internal carotid artery until it blocked the origin of the middle cerebral artery. Control sham-operated animals were subjected to the same surgical procedure without insertion of the filament. Achievement of ischemia was confirmed by monitoring regional cerebral blood flow in the area of the right MCA. Cerebral blood flow was monitored through a disposable microtip fiber optic probe (diameter 0.5mm) connected through a Master Probe to a laser Doppler computerized main unit (PF5001; Perimed, Sweden) and analyzed using PSW Perisoft 2.5. Rectal temperature was maintained at 37±0.5°C with a thermostatically controlled heating pad and lamp. All surgical procedures were performed under an operating stereomicroscope. After 60 min MCA occlusion, mice were reanesthetized and the filament was withdrawn in order to restore blood flow. For siRNAs administration, animals were placed on a stereotaxic frame, and siRNA was injected through a 27-g stainless steel guide cannula implanted into the right lateral ventricle using the stereotaxic coordinates from the bregma: +0.6mm caudal, -1.6mm lateral and 2.3mm below the dura. siRNAs administration was performed (1ml, 10μM) 24 hours before ischemia induction. Depending on the experiment, all mice were euthanized at 6, 12 and 24 hours after tMCAO, and the temporoparietal cortices were harvested for qRT-PCR, ChIP, and Western Blot analysis, Iron and 4-HNE assay.

#### Immunostaining and confocal immunofluorescence

Confocal immunofluorescence procedures were performed as previously described 32, 40. In particular, mice were anesthetized with 1.5% sevoflurane and 98.5% O2, and transcardially perfused with saline solution containing 0.01 ml heparin, followed by 60 ml of 4% paraformaldehyde in saline solution. Brains were rapidly removed on ice and fixed overnight at +4°C and cryoprotected in 30% sucrose in 0.1 M phosphate buffer (PB) with 0.02% sodium azide for 24 h at +4°C. Brains were cut on a cryostat into 40 µm coronal sections in rostral-caudal direction. Afterwards, free floating sections were incubated with blocking solution (0.5% milk, 10% FBS, 1% BSA) in PBS for 1.5 h. The primary antibodies were the following: anti-NeuN (1:1000, Millipore, Milan, Italy; ABN78), anti-Sp1 (1:500, Santa Cruz, Dallas, Texas USA; sc-17824), anti-HIF-1alpha (1:500, Santa Cruz, Dallas, Texas USA; sc-13515), anti-TfR1 (1:500, Santa Cruz, Dallas, Texas USA; sc-65882), anti-GPX4 (1:500, Millipore, Milan, Italy; SAB4300725), anti-HDAC9 (1:500, Santa Cruz, Dallas, Texas USA; sc-28732), anti-GFAP (1:1000 Millipore, Milan, Italy; MAB360), anti-IBA1 (1:1000, Wako, Chuo-Ku, Tokyo; PN019-19741). The sections were then incubated with the corresponding florescent-labeled secondary antibodies, Alexa 488/Alexa 594 conjugated antimouse/antirabbit IgGs (715-545-150, 711-585-152, Jackson Immuno Research Baltimore, PA). Nuclei were counterstained with Hoechst (Sigma-Aldrich, Milan, Italy). Images were observed using a Zeiss LSM700 META/laser scanning confocal microscope (Zeiss, Oberkochen, Germany). Single images were taken with a resolution of 1024 × 1024.

#### Fluorescence intensity analysis

The fluorescence of proteins HDAC9, HIF1, TfR1, SP1, GPX4 has been quantified in terms of pixel intensity on tissue sections at the level of the cortex by using Image J software, as previously described 41, 42. Images from the same areas of brain cortex were compared. Results were expressed in arbitrary units, n = 3 mice per treatment groups.

### Statistical analysis

Data were analyzed by GraphPad Prism 5 software (Graph Pad Software, Inc.). All bars in the figures represent the mean ± S.D. Statistical differences between two experimental groups were analyzed with the Student t-test. Statistically significant differences between more than two experimental groups were evaluated by one-way ANOVA, followed by Turkey's multiple comparison test.

## Results

### OGD/Rx induced an increase of HDAC9, TfR1 and a reduction of GPX4 in neuronal-like SH-SY5Y cells. Silencing of HDAC9 reverted OGD/RX-induced TfR1 up-regulation and GPX4 down-regulation and limited OGD/RX-induced cell death

**Figure 1:**
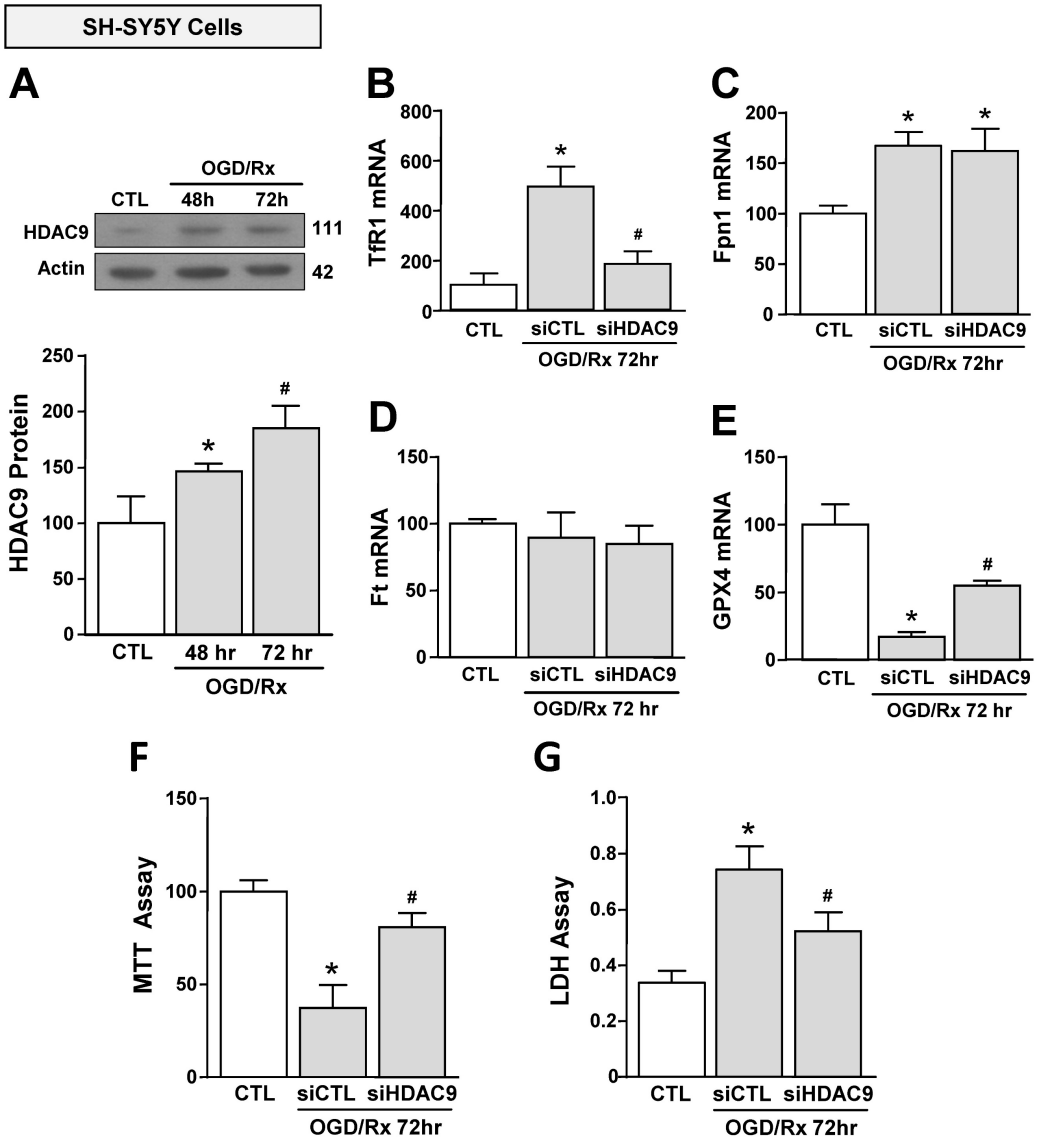
**Effects of siHDAC9 on cell death and expression of ferroptotic genes in SH-SY5Y cells after OGD/Rx. A**, HDAC9 protein expression at 48h or 72h after OGD/Rx. (n = 5). *, p ≤ 0.05 versus control under normoxic conditions (CTL); #, p ≤ 0.05 versus 48h after OGD/Rx. **B-E**, Effects of siHDAC9 or scrambled siRNA on gene expression of TfR1 (B), Fpn1 (C), Ft (D) and GPX4 (E) after OGD/Rx 72h. (n = 3); *, p ≤ 0.05 versus control (CTL); #, p ≤ 0.05 versus siCTL+. **F-G**, Viability measured with MTT assay (F) and cell death measured as LDH release (G) in cells treated with siRNA for HDAC9 or scrambled at 72h after OGD/Rx. (n = 4). *, p ≤ 0.05 versus control (CTL); #, p ≤ 0.05 versus siCTL. Bars represent mean ± SD.

**Figure 2:**
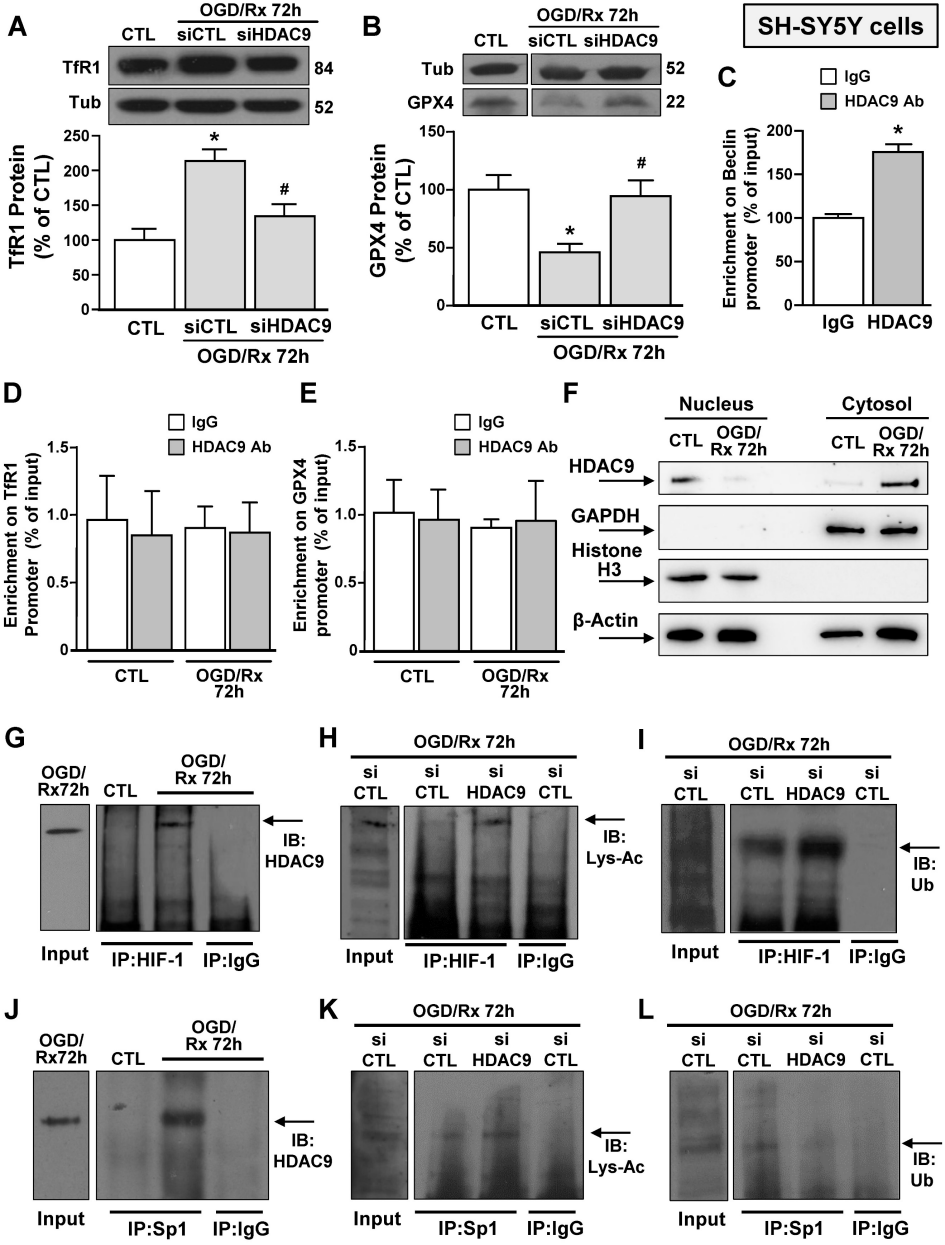
** Effects of OGD/Rx to modulate HDAC9 nucleus/cytosol translocation and of siHDAC9 to revert OGD/Rx-induced HIF-1 and Sp1 deacetylation, in neuronal-like SH-SY5Y cells. A-B,** Representative Western blots with quantification of TfR1 (A) and GPX4 (B) protein expression in SH-SY5Y cells treated with siRNA for HDAC9 or scrambled control at 72h after OGD/Rx. Bars represent mean ± SD (n = 5); *, p ≤ 0.05 versus control under normoxic conditions (CTL); #, p ≤ 0.05 versus siCTL under OGD/Rx conditions. **C,** Chromatin immunoprecipitation (ChIP) on Beclin promoter performed in NSC34 cells using anti-HDAC9 or anti-IgG used as negative control under control conditions or at 72 h after OGD/Rx. Bars represent mean ± SD (n = 4). **D-E,** Chromatin immunoprecipitation (ChIP) on TfR1 (C) and GPX4 (D) promoter performed in SH-SY5Y cells using anti-HDAC9 or anti-IgG used as negative control under control conditions or at 72 h after OGD/Rx. Bars represent mean ± SD (n = 5). **F,** Representative Western blot of HDAC9 protein levels in nucleus and cytosol of SH-SY5Y cells under control condition and at 72 h after OGD/Rx. Anti-GAPDH and anti-histone H3 (H3) were used to verify the purity of the cytoplasmic and nuclear fractions, respectively. (n = 5). **G-L,** Representative immunoprecipitation Western blot showing the interaction between HDAC9 and HIF-1 (G) or Sp1 (J) in SH-SY5Y cells under control conditions or at 72h after OGD/Rx; association between HIF-1 and lysine-acetylated (Lys-Ac) (H) or ubiquitine (Ub) (I) in SH-SY5Y cells treated with siHDAC9 or scrambled at 72h after OGD/Rx; association between Sp1 and Lys-Ac (K) or Ub (L) in SH-SY5Y cells treated with siHDAC9 or scrambled at 72h of OGD/Rx. The Input samples in panels G and J are SH-SY5Y cells subjected to OGD/Rx 72h, whereas in panels H, I, K and L are SH-SY5Y cells exposed to OGD/Rx 72h after siCTL transfection.

We investigated the effect of OGD/Rx on HDAC9 protein expression in cell lines participating in the neurovascular unit (NU) involved in the stroke pathophysiology 43. HDAC9 protein levels were increased in the neuronal-like SH-SY5Y cells at 48 and 72h after OGD/Rx (Fig. 1A). Furthermore, among the gene products involved in iron homeostasis, TfR1 and Fpn1 mRNA were increased at 72h (Fig. 1B, C), whereas Ft mRNA was unaffected (Fig. 1D). In addition, mRNA levels of the ferroptotic defense enzyme GPX4 were reduced (Fig. 1E). More relevantly, siHDAC9 reverted at transcriptional and translational levels TfR1 overexpression (Fig. 1B and 2A), GPX4 downregulation (Fig. 1B and 2B) and cell damage induced by OGD/Rx (Fig. 1F and G).

The regulation of the ferroptotic pathway induced by OGD/Rx on HDAC9 expression was different in the other cells belonging to the NU, such as glial-like U87 and endothelial-like bEND3 cells, as compared to SH-SY5Y cells. Indeed, OGD/Rx induced: (i) an increase of HDAC9 (Supplementary Fig. 1A), (ii) an up-regulation in TfR1 expression in bEND3 cell line, but not in U87 cells (Supplementary Fig. 1D), (iii) no variation in Fpn1 in bEND3 and U87 cells (Supplementary Fig. 1E), (iv) an increase or a reduction of Ft in U87 and bEND3 cells, respectively (Supplementary Fig. 1F), and (v) a downregulation of GPX4 in bEND3 and U87 cells (Supplementary Fig. 1G). Interestingly, siHDAC9 although reduced cell damage induced by OGD/Rx in U87 and bEND3 cells (Supplementary Fig. 1B and C), it did not revert the mRNA variations of GPX4 and of genes involved in iron homeostasis (Supplementary Fig. 1G). These results suggest that HDAC9 can promote cell death by different players in neuronal and non-neuronal cells. Notably, siHDAC9 significantly reduced HDAC9 mRNA expression in human 37 and mouse cell line (Supplementary Fig. 3A).

### HDAC9 protein levels increased in the cytoplasm but not in the nucleus of neuronal-like SH-SY5Y cells upon exposure to OGD/RX

Since after OGD/Rx, the siHDAC9 reverted the TfR1 increase and GPX4 reduction (Fig. 2A, B), we investigated whether HDAC9 might modify its relative enrichment to the human promoter sequences of these two genes. Chromatin immunoprecipitation assay between HDAC9 antibody and GPX4 or TfR1 promoter sequences of OGD/Rx-treated SH-SY5Y cells showed that no alterations in DNA enrichment was detected after OGD/Rx, as compared to control IgG (Fig. 2D, E). However, HDAC9 was able to bind to the promoter sequence of its known target gene Beclin 38, as compared to control IgG (Fig. 2C).

Since OGD/Rx did not modify the relative enrichment of HDAC9 on GPX4 or TfR1 promoter sequences, we investigated whether this lysine-deacetylation enzyme might be confined and accumulated in the cytosolic compartment after OGD/Rx. Results showed that HDAC9, upon OGD/Rx exposure, selectively increased in cytoplasm, but not in the nucleus, suggesting that this lysine-deacetylation enzyme modulates TfR1 and GPX4 gene expression by regulating transcription factors in the cytosol compartment (Fig. 2F).

### siHDAC9 reverted OGD/Rx-induced HIF-1 and Sp1 deacetylation, determining deubiquitination for HIF-1 and a ubiquitination for Sp1 in neuronal-like SH-SY5Y cells

We investigated the possible interaction between HDAC9 and transcriptional factors involved in stroke pathophysiology, that are able to activate in a sequence specific manner their target genes TfR1 22 and GPX4 26, such as HIF-1 and Sp1 in SH-SY5Y cells after OGD/Rx. Both Sp1 and HIF-1 were found singly bound to HDAC9 (Fig. 2G and J) and deacetylated (Fig. 2H and K), as revealed by immunoprecipitation assay with an anti-Sp1 or anti-HIF-1 antibody, followed by immunoblotting with an anti-HDAC9, or anti-acetyl lysine antibody. On the other hand, ubiquitination signals were reduced on HIF-1 (Fig. 2I) and increased on Sp1 (Fig. 2L). Accordingly, SH-SY5Y cells transfected with siHDAC9 displayed after OGD/Rx a reduction in: (i) the deacetylation of both transcription factors (Fig. 2H and K); (ii) the deubiquitination of HIF-1 (Fig. 2HI); and (ii) the ubiquitination of Sp1 (Fig. 2L).

**Figure 3:**
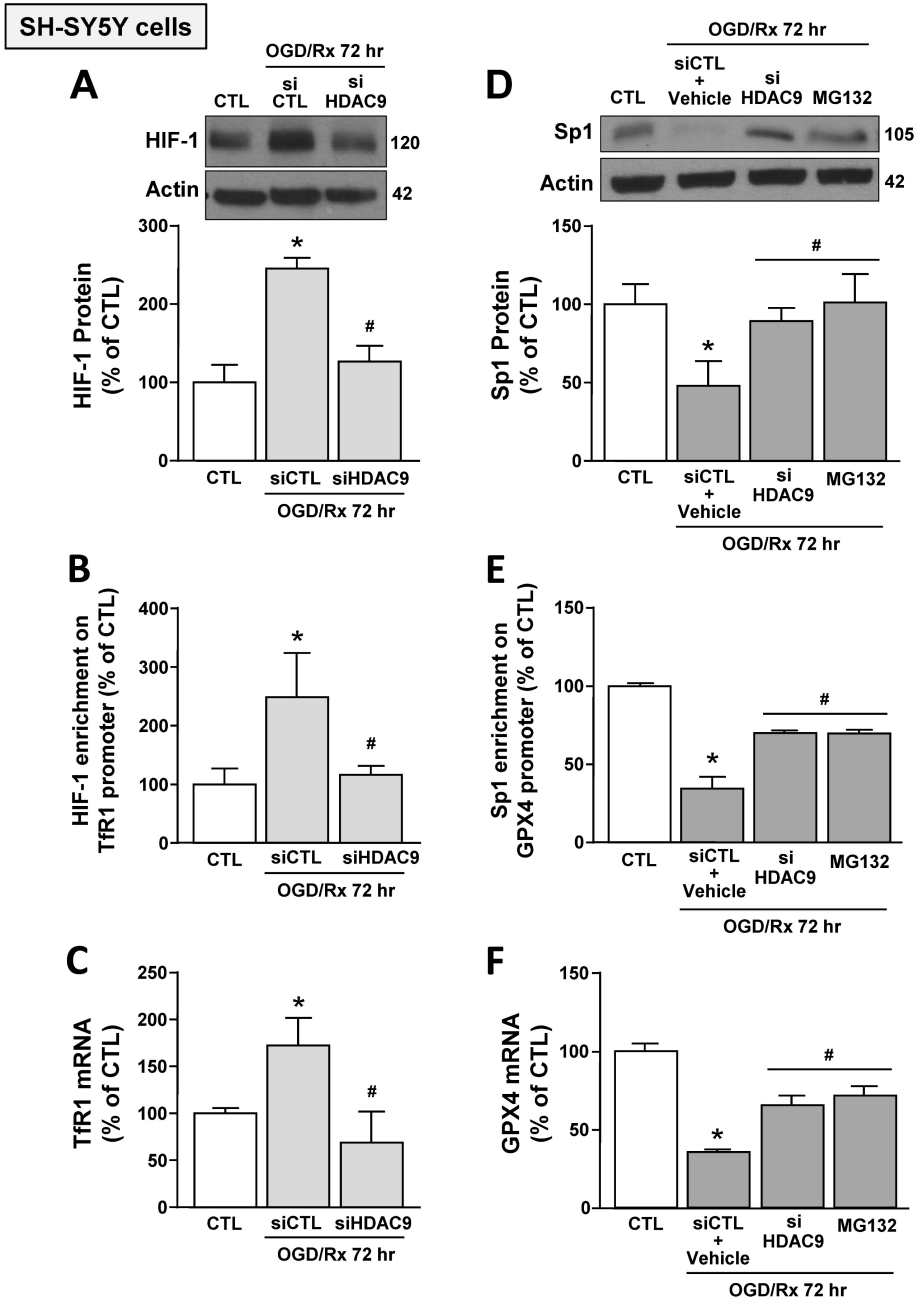
** Effects of HDAC9 silencing on HIF-1-dependent TfR1 upregulation and Sp1-dependent GPX4 reduction in SH-SY5Y cells subjected to OGD/Rx. A, D,** Representative western blot and quantification of HIF-1 (A) or Sp1 (D) protein expression in SH-SY5Y cells treated with siHDAC9 or scrambled control (siCTL) or with MG132 (10 μM) at 72h after OGD/Rx (n = 3/5). *, p ≤ 0.05 versus normoxic control (CTL); #, p ≤ 0.05 versus siCTL or siCTL plus vehicle in hypoxic conditions. **B, E,** Chromatin immunoprecipitation performing by using antibodies for HIF-1 (B) or for Sp1 (E), and primers for TfR1 and GPX4 promoter sequences, respectively, in SH-SY5Y cells under normoxic conditions or treated with siCTL, siHDAC9, or with MG132 (10 μM) and then subjected to OGD/Rx 72h (n = 3/5). **C, F,** mRNA levels of TfR1 (C) and GPX4 (F) in SH-SY5Y cells treated with siCTL or siHDAC9 or MG132 at 72h after OGD/Rx. (n = 3); *, p ≤ 0.05 versus normoxic condition (CTL); #, p ≤ 0.05 versus siCTL or siCTL + vehicle in hypoxic conditions. Bars represent mean ± SD.

Furthermore, siHDAC9 transfection in SH-SY5Y cells after OGD/Rx counteracted: (i) HIF-1 protein increase and Sp1 reduction (Fig. 3A,D); (ii) the increased binding of HIF-1 on TfR1 gene and the reduced binding of Sp1 on GPX4 gene (Fig. 3B,E); and (iii) TfR1 mRNA increase and GPX4 mRNA reduction (Fig. 3C,F). In addition, pre-treatment with the proteasome inhibitor MG132 (1 μM) in OGD/Rx cells prevented: (i) Sp1 protein downregulation (Fig. 3D); (ii) the reduction of Sp1 binding on GPX4 promoter sequence (Fig. 3E); and (iii) the downregulation of GPX4 (Fig. 3F).

These results indicated that OGD/Rx induced the binding of HDAC9 with either HIF-1 and Sp1, and this binding let to the deacetylation of these two transcription factors causing: (i) an increase in HIF-1 protein levels, its binding to TfR1 gene, and in TfR1 gene transcription; and (ii) a reduction in Sp1 protein levels, its binding on GPX4 promoter, and in GPX4 transcription.

### siHDAC9 attenuated ferroptotic neuronal damage by blocking HIF-1-dependent TfR1 increase and Sp1-dependent GPX4 reduction in cortical neurons subjected to OGD/Rx

**Figure 4:**
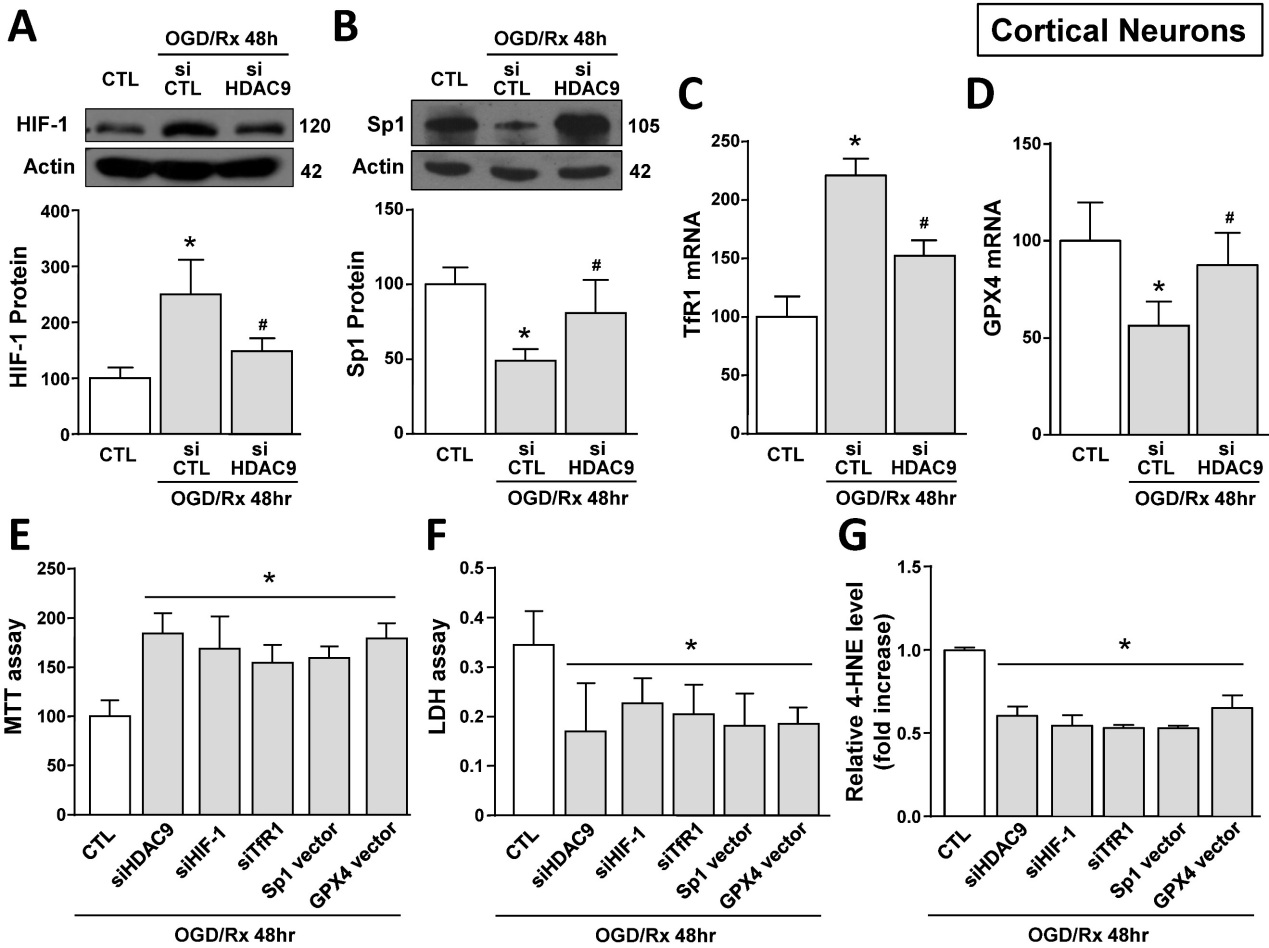
** Effect of HDAC9 silencing on HIF-1 and Sp1 protein expression and on viability of primary cortical neurons exposed to OGD/Rx. A, B,** Representative Western blot and quantification of HIF-1 (A) or Sp1 (B) protein expression in primary cortical neurons under normoxic conditions or treated with siCTL or siHDAC9 and subjected to OGD/Rx 48h (n = 5). *, p ≤ 0.05 versus normoxic control (CTL); #, p ≤ 0.05 versus siCTL + OGD/Rx 48hr.** C, D,** mRNA levels of TfR1 (C) and GPX4 (D) in cells under normoxic conditions or treated with siCTL or siHDAC9 and subjected to OGD/Rx 48h (n = 3); *, p ≤ 0.05 versus normoxic control (CTL); #, p ≤ 0.05 versus siCTL + OGD/Rx 48hr. **E-G,** Effects of silencing of HDAC9, HIF-1, TfR1 or overexpression of Sp1 or GPX4 on cell viability, as measured by MTT assay (E), LDH (F) and 4-HNE (G) release in primary cortical neurons subjected to OGD/Rx 48h (n = 5); *, p ≤ 0.05 versus siCTL plus mock (CTL). Bars represent mean ± SD.

The role of HDAC9 in the ferroptotic pathway was also confirmed in primary cortical neurons exposed to OGD/Rx. Indeed, 24 and 48 hours after OGD/Rx exposure, HDAC9 mRNA and protein expression was significantly increased as compared to normoxic conditions (Supplementary Fig. 2A and B). In particular, transfection of siHDAC9 in cortical neurons exposed to OGD/Rx reverted: (i) the increase in HIF-1 protein and in TfR1 mRNA levels (Fig. 4A,C) and (ii) the reduction in Sp1 protein and GPX4 mRNA levels (Fig. 4B,D). Notably, HIF-1 and Sp1 mRNAs were not affected by OGD/Rx stimuli alone or in combination with siHDAC9 (Supplementary Fig. 2C and D).

Then we studied the role of HDAC9 on HIF-1 and TfR1 upregulation and on Sp1 and GPX4 downregulation in OGD/Rx-induced ferroptotic cell death. In particular, we silenced HDAC9, HIF-1, TfR1 or overexpressed Sp1 or GPX4 by plasmid transfection. The efficiency transfection of siHIF-1 and of Sp1 vector were previously reported 30, siTfR1 significantly decreased TfR1 expression by 62% (Supplementary Fig. 3C), whereas GPX4 vector significantly increased GPX4 expression by 90% (Supplementary Fig. 3D), as compared to respective control group. Intriguingly, transfection of siHDAC9, siHIF-1, siTfR1, Sp1 vector or GPX4 vector significantly reduced OGD/Rx-induced cell death, as revealed by MTT and LDH assays (Fig. 4E and F). Furthermore, to confirm the role of ferroptosis, we measured the levels of the ferroptotic marker 4-HNE, an aldehyde secondary product of lipid peroxidation 44 in the medium of neurons exposed to OGD/Rx. In particular, 4-HNE levels were reduced in OGD/Rx-treated neurons transfected with siHDAC9, siHIF-1, siTfR1, or with vectors for Sp1 or GPX4, as compared to OGD/Rx control neurons (Fig. 4G). Furthermore, since HDAC1 and 2, belonging to class I HDACs, and HDAC4 and 5, belonging to class II HDACs, increase in an *in vitro* model of stroke 30, 39, we investigate the specificity of HDAC9, among HDACs isoforms, in the modulation of TfR1 and GPX4 after ODG/Rx. Results showed that in cortical neurons OGD/Rx exposure significantly increased HDAC 1, 2, 4 and 5 protein levels (Supplementary Fig. 4A-D), but silencing of HDAC1, 2, 4 or 5 did not affect TfR1 mRNA increase (Supplementary Fig. 4E) or GPX4 mRNA reduction (Supplementary Fig. 4F).

### HDAC class IIa inhibitor MC1568 attenuated OGD/Rx-induced ferroptotic damage in cortical neurons by blocking HDAC9 increase

**Figure 5:**
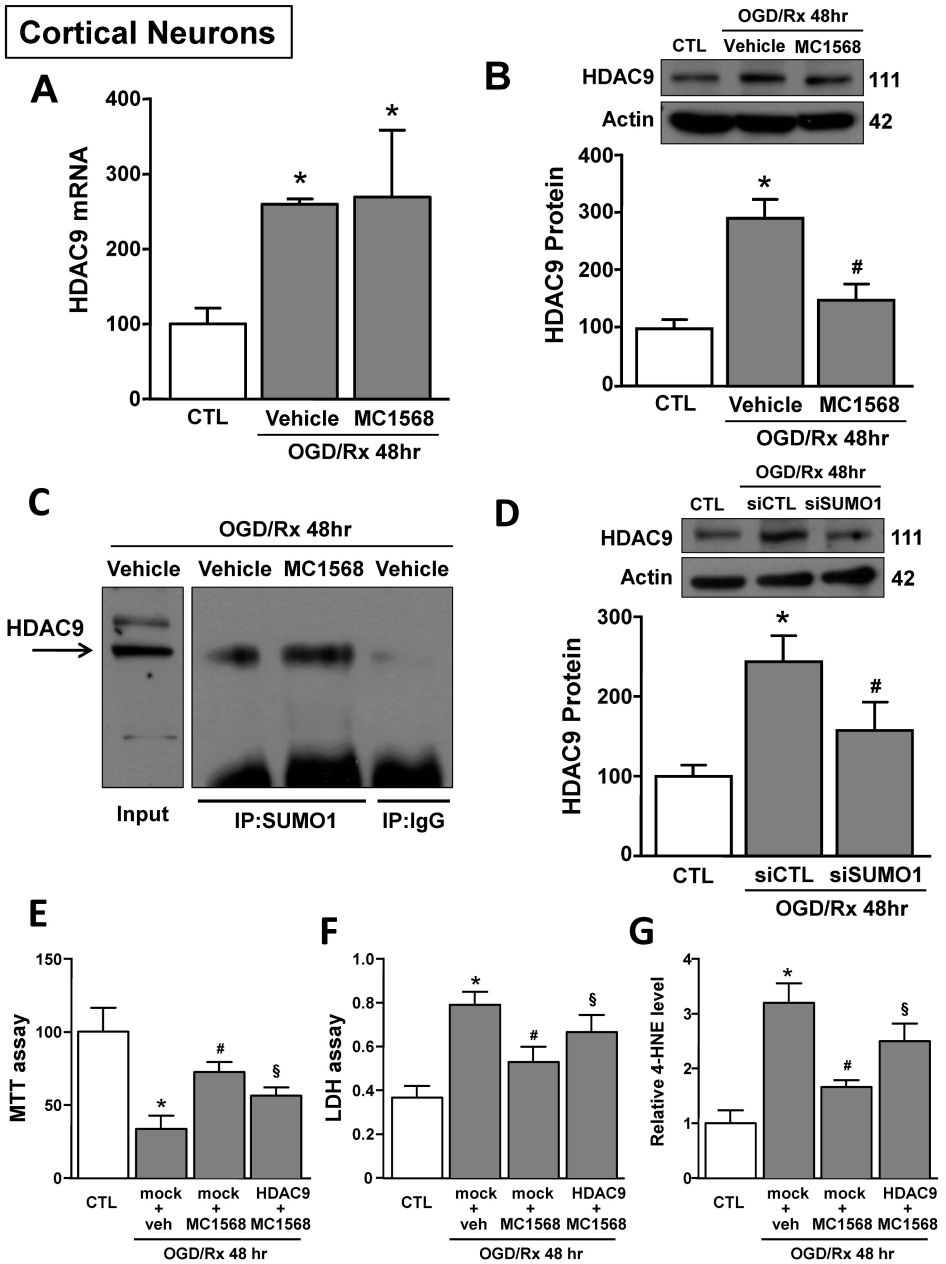
** Effects of MC1568 on HDAC9 mRNA and protein levels and the role of SUMO1 in MC1568-induced HDAC9 downregulation in cortical neurons subjected to OGD/Rx. A-B,** Effect of MC1568 on HDAC9 mRNA (A) and protein (B) levels at 48 h of OGD/Rx (n = 3/5); *, p ≤ 0.05 versus control under normoxic conditions (CTL); #, p ≤ 0.05 versus vehicle under OGD/Rx 48h. **C,** Representative Immunoprecipitation-Western blot showing the physical interaction between SUMO1 and HDAC9 at 48 hr after OGD/Rx in primary cortical neurons treated with MC1568 or vehicle. **D,** Effect of siRNA for SUMO1 on HDAC9 protein expression at 48 hr after OGD/Rx (n = 5); *, p ≤ 0.05 versus control under normoxic conditions (CTL); #, p ≤ 0.05 versus siCTL after OGD/Rx 48 hr. **E-G,** Effects of MC1568 on cell viability, as measured by MTT assay (E), LDH (F) and 4-HNE (G) release, in primary cortical neurons at 48 hr after OGD/Rx (n = 5); *, p ≤ 0.05 versus control under normoxic conditions (CTL); #, p ≤ 0.05 versus mock + vehicle at 48hr after OGD/Rx; §, p ≤ 0.05 versus mock+MC1568 at 48hr after OGD/Rx. Bars represent mean ± SD.

The HDAC class IIa inhibitor MC1568 induced neuroprotection after OGD/Rx by blocking HDAC4 and HDAC5 upregulation 39. We investigated whether the neuroprotective mechanism by which MC1568 induced neuroprotection was also due to HDAC9 counteraction. MC1568 significantly prevented HDAC9 protein upregulation at 48hr after OGD/Rx, but did not modify its mRNA level (Fig. 5A and B). It has been described that MC1568 induces HDAC4 reduction by increasing its specific sumoylation and consequently protein degradation 45. As shown in Figure 5C, SUMO1 bound to HDAC9 in cortical neurons after MC1568 treatment. Furthermore, siRNA against SUMO1 counteracted MC1568-induced HDAC9 protein reduction in OGD/Rx-treated neurons (Fig. 5D). Moreover, the transfection of a vector overexpressing HDAC9 39, blocked MC1568-induced neuroprotection in cortical neurons exposed to OGD/Rx, as revealed by MTT, LDH and 4-HNE assays (Fig. 5E-G).

### siHDAC9 blocked tMCAO-induced ferroptotic neuronal death

**Figure 6:**
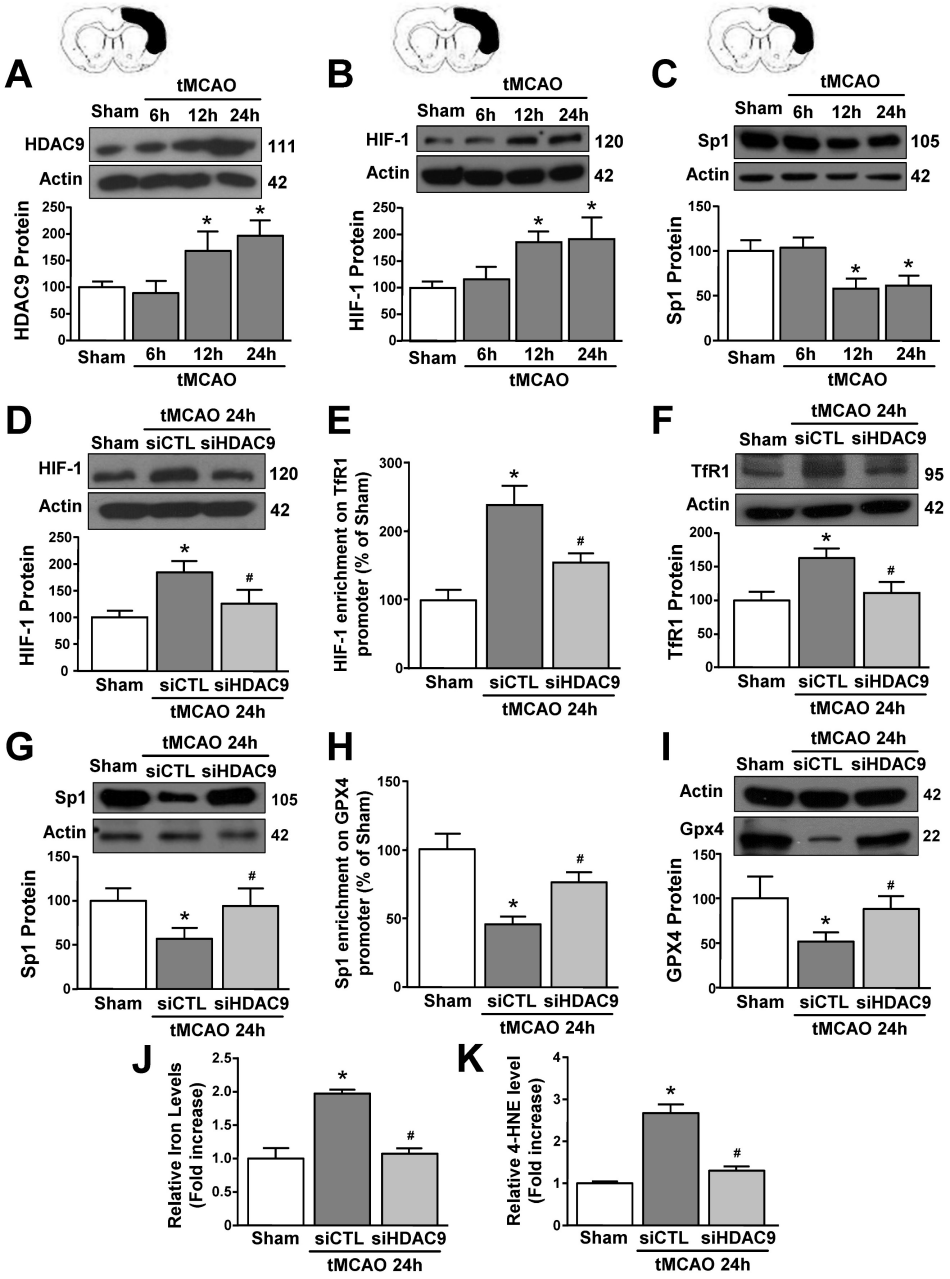
** HDAC9 knockdown alleviates ferroptotic neuronal death by blocking the increase of HIF-1 binding on TfR1 gene and by enhancing Sp1 binding on GPX4 gene in the periischemic temporoparietal cortex of tMCAO mice. A-C,** Representative Western blot and quantification of HDAC9 (A), HIF-1alpha (B), and Sp1 (C) protein levels in temporoparietal cortex of mice after tMCAO or sham operated animals (Sham) (n = 5/6); *, p ≤ 0.05 versus Sham. **D, G,** Representative Western blot and quantification of HIF-1alpha (D) and Sp1 (G) protein levels in temporoparietal cortex of sham operated mice or mice subjected to 24h of tMCAO and treated with siHDAC9 or scrambled siRNA (siCTL) (n=5); *, p ≤ 0.05 versus Sham; #, p ≤ 0.05 versus siCTL after 24h of tMCAO. **E, H,** Chromatin immunoprecipitation (ChIP) of HIF-1 alpha on TfR1 promoter (E) and Sp1 on GPX4 promoter (H) performed in temporoparietal cortex of sham operated mice or mice subjected to 24h of tMCAO and treated with siHDAC9 or siCTL. Anti-IgG antibody has been used as negative control (n = 3); *, p ≤ 0.05 versus Sham, #, p ≤ 0.05 versus siCTL after 24h of tMCAO. **F, I,** Representative Western blot and quantification of TfR1 (F) and GPX4 (I) protein levels in temporoparietal cortex of sham operated mice or mice subjected to 24h of tMCAO and treated with siHDAC9 or siCTL (n = 5); *, p ≤ 0.05 versus Sham; #, p ≤ 0.05 versus siCTL after 24h of tMCAO. J, K, quantification of relative Fe^2+^ (J) and 4-HNE (K) levels in temporoparietal cortex of sham operated mice or mice subjected to 24h of tMCAO and treated with siHDAC9 or scrambled siRNA (n = 5); *, p ≤ 0.05 versus Sham; #, p ≤ 0.05 versus siCTL after 24h of tMCAO. Bars represent mean ± SD.

To validate results obtained *in vitro* we explored the role of HDAC9 in the ferroptotic pathway in mice subjected to transient middle cerebral artery occlusion. An increase in HDAC9 and HIF-1 protein levels and a reduction in Sp1 protein occurred at 12 and 24 hours after tMCAO, as compared to sham-operated animals (Fig. 6A-C). Accordingly, confocal double immunofluorescence experiments performed in the periischemic temporoparietal cortex region, revealed that HDAC9 (Supplementary Fig. 5 and 7A), HIF-1 (Supplementary Fig. 6A and 7B) and TfR1 (Supplementary Fig. 6B and 7C) immunosignal significantly increased whereas Sp1 (Supplementary Fig. 6C and 7D), and GPX4 expression significantly decreased (Supplementary Fig. 6D and 7E) in the cortical layer, as compared to the respective sham-operated animals.

Notably, tMCAO-induced HIF-1 and TfR1 increase did not occur in GFAP-positive cells (Supplementary Fig. 8), but occurred either in NeuN- (Supplementary Fig. 5) and IBA1-positive cells (Supplementary Fig. 9). Furthermore, icv administration of siHDAC9 that reduced its mRNA levels of 58% (Supplementary Fig. 3B), significantly prevented tMCAO-induced up-regulation of HIF-1 and down-regulation of Sp1 protein levels (Fig. 6D and G). In accordance, confocal double immunofluorescence experiments showed that silencing of HDAC9 caused a significant reduction in HIF-1 and TfR1 immunosignals (Supplementary Fig. 6A,B and 7B,C) and a significant increase of Sp1 and GPX4 (Supplementary Fig. 6C,D and 7D,E) in the ischemic cortical layer.

Importantly, siHDAC9 counteracted the increase in HIF-1 binding on TfR1 promoter and, thus, TfR1 mRNA increase after stroke (Fig. 6E and F). By contrast, siHDAC9 reduced Sp1 binding on GPX4 promoter and, thus, reduced the transcription of GPX4 gene (Fig. 6 H and I).

Furthermore, levels of iron and 4-HNE were found to be significantly increased in tMCAO group (Fig. 6J and K). Intriguingly, siHDAC9 significantly counteracted the increase of iron and 4-HNE levels after tMCAO (Fig. 6J and K).

## Discussion

This paper showed for the first time that HDAC9 is involved in the activation of ferroptotic cell death in *in vitro* and *in vivo* models of stroke. Indeed, brain ischemia induced an up-regulation in HDAC9 protein levels that binds to the two transcription factors HIF-1 and Sp1 causing their deacetylation. This process determined: (1) an increase in HIF-1 protein levels, which raises the expression of its pro-ferroptotic target gene TfR1; (2) a downregulation of Sp1 and its anti-ferroptotic target gene GPX4. Both transcriptional events contribute to the activation of the HDAC9-mediated ferroptotic cell death in stroke.

The contribution of HDAC9 in ferroptotic cell death after stroke was demonstrated by the results showing that HDAC class IIA inhibitor, MC1568, known to also inhibit HDAC9 46, reduced ferroptotic neuronal death after OGD/Rx, whereas HDAC9 overexpression counteracted the neuroprotective effect of MC1568. In addition, the specific role of HDAC9 was determined by the silencing of this deacetylating enzyme that significantly reduced two ferroptotic markers, such as iron overload and 4-HNE, in temporoparietal cortex after stroke. Furthermore, silencing of HDAC9 also prevented HIF-1 increase, Sp1 decrease, and consequently, the modulation of their target genes TfR1 and GPX4 in neurons in in vitro and in vivo models of stroke as demonstrated by Western blot and immunohistochemistry experiments. Moreover, silencing of HDAC9, besides preventing HIF-1 and Sp1 deacetylation after OGD/Rx, it also prevented deubiquitination of HIF-1 and the ubiquitination of Sp1. Thereafter, upon these post-traslational modifications, HIF-1 avoids degradation and increases its protein levels, whereas Sp1 is committed for the proteasome pathway. In fact, HDAC9 showed not to affect transcriptional levels of HIF-1 and Sp1 as revealed by qRT-PCR in primary cortical neurons after OGD/Rx, whereas the proteasome inhibitor MG132 prevented Sp1 protein downregulation. In accordance with these results, it was previously reported that the deacetylation of HIF-1 is associated to its deubiquitination and protein stability, thus increasing the protein levels of the hypoxic factor 47. Moreover, HIF-1 is rapidly degraded by the ubiquitin-proteasome system under normoxic conditions, as it occurs during reoxydative phase, whereas it is not ubiquitinated under hypoxic conditions without reoxygenation 48, 49. On the contrary it was already reported that Sp1 is deacetylated during oxidative stress as it occurs during the reoxygenation phase 50.

Another intriguing finding emerging from these results is that HDAC9, beyond deacetylating histones in nuclei, it was also found to deacetylate HIF-1 and Sp1 in the cytosolic compartment after OGD/Rx. These results were in accordance with other authors that found other members of class IIa HDACs to deacetylate proteins in the cytosolic compartment, a compartment in which they can be restricted by a Calmodulin Kinase II phosphorylation post-translational modifications 51.

It is worth mentioning that TfR1 and GPX4 mRNA levels were regulated by HDAC9 in a cell-type specific manner, since, although HDAC9 was found to be increased in neuronal, glial-like U87 and endothelial-like eBEND3 cells, the HDAC9-dependent modulation of TfR1 and GPX4 expression selectively occurred in primary cortical neurons exposed to OGD/Rx, and in the perischemic cortical regions of mice subjected to tMCAO. Furthermore, both HIF-1 protein increase and Sp1 protein decrease occurred in temporoparietal cortex of mice starting from 12h after tMCAO; a time-point in which HDAC9 protein expression peaked. Coherently, the transcriptional effects on the downstream genes of this pathway, TfR1 and GPX4, were observed later, at 24 hours after tMCAO, a time-point compatible with a transcriptional regulation by HIF-1 and Sp1, respectively. Interestingly, both the increase in HIF-1/TfR1 pathway and the decrease in Sp1/GPX4 pathway lead to a common endpoint that is represented by ferroptotic cell death. Indeed, silencing of HIF-1 or its target gene TfR1 caused a reduction of ferroptotic neuronal death after OGD/Rx. In addition, similar results were also obtained overexpressing either Sp1 or its target gene GPX4 under same experimental conditions.

Noteworthy, the predictive scores for mouse, rat, and human HIF-1 binding sites are 96%, 94%, and 99% respectively (JASPAR 2023, TF profile matrix ID: MA0259.1) and for Sp1 binding sites are 91%, 93% and 92% respectively (JASPAR 2023, matrix ID: MA0079.3). Interestingly, the position of all the aforementioned sequences, on TfR1 and GPX4 mouse, rat, and human promoters are conserved. This high percentage of homology between mouse, rat, and human TfR1 and GPX4 promoter sequences indicates the relevance of our results obtained in animal models of stroke to human brain ischemia. In accordance with the neurodetrimental role of HIF-1 in stroke, other reports show that the prevention of the degradation of the hypoxic factor, by blocking HIF-1 prolyl hydroxylases, improved functional recovery and neuronal survival in mouse striatum after ischemic cerebral hemorrhage 52. On the other hand, the neuroprotective role of Sp1 to reduce ferroptosis was also previously observed by other authors by blocking oxidative stress in cortical neurons 53, and by counteracting GPX4 reduction after hemorrhagic stroke 26.

It should be mentioned that some reports showed an overexpression in HDAC9 protein levels in cerebral microvessel endothelial cells after stroke where this deacetylating enzyme contributes to the production of pro-inflammatory mediators 12. For these reasons it was hypothesized that HDAC9 might promote apoptotic neuronal death 12. However, our results showed that siHDAC9 did not prevent the activation of the pro-apoptotic marker caspase 3 in primary cortical neurons after OGD/Rx, thus supporting the hypothesis that HDAC9 selectively induces ferroptotic neuronal death.

In accordance with this hypothesis, the selective class IIa HDACs inhibitor, MC1568, caused the binding of HDAC9 to SUMO1 with a consequent degradation of this deacetylation factor in cortical neurons after OGD/Rx, as far as to other HDAC class IIa members 29, 45, and exerts a neuroprotective effect after stroke 39. However, although it was reported that HDAC4 represents a player for MC1568-dependent neuroprotection 39, present results showed that this effect was also mediated by the sumoylation-dependent degradation of HDAC9.

Intriguingly, supporting the neurodetrimental role of HDAC9, polymorphisms of this gene that increase its expression levels are associated to an increased risk of stroke occurrence 8.

Another aspect that should be considered is that it has also been reported that the pharmacological inhibition of other HDACs isoforms, including HDAC class I, might also exert a role in neuronal ferroptosis in stroke 16. However, although we found that HDAC1, 2, 4 and 5 isoforms increased in primary cortical neurons after OGD/Rx 30, 54, here we also found that the silencing of these epigenetic factors did not affect TfR1 and GPX4 gene expression. These data reinforce the hypothesis that HDAC9 selectively promotes ferroptotic neuronal death by means of TfR1 and GPX4. On the other hand, we cannot exclude that HDAC9 could also regulate other players participating in iron homeostasis and/or ferroptosis, including divalent metal transporter 1 (DMT1) and Six Transmembrane Epithelial Antigen of Prostate 3 (STEAP3) proteins 55.

Collectively, the results of the present study suggest that stroke induced an increase in HDAC9 in the cytosol of neurons activating the pro-ferroptotic pathway HIF-1/TfR1 and reduces the anti-ferroptotic pathway Sp1/GPX4.

Therefore, the development of drugs able to specifically inhibit HDAC9 overexpression or activity could represent a new pharmacological strategy to reduce ferroptosis and brain damage after stroke.

## Supplementary Material

Supplementary figures.
