# Supplementary material for: Stroke by inducing HDAC9-dependent deacetylation of HIF-1 and Sp1, promotes TfR1 transcription and GPX4 reduction, thus determining ferroptotic neuronal death
Source: Int J Biol Sci. 2023 May 11;19(9):2695–710. doi: 10.7150/ijbs.80735 (PMC10266075; doi:10.7150/ijbs.80735)
Supplement: Supplementary file 1 — Supplementary figures. [file ijbsv19p2695s1.pdf]

Supplemental Figure Legends

Suppl Fig 1. Effects of siHDAC9 on cell death and expression of ferroptotic genes in U87 and bEND3 cells after OGD/Rx.

A. HDAC9 protein expression at 48h or 72h after OGD/Rx. *, $p \leq 0.05$ versus control under normoxic conditions (CTL). **B-C,** Viability measured with MTT assay (B) and cell death measured as LDH release (C) in cells treated with siRNA for HDAC9 or scrambled at 72h after OGD/Rx. *, $p \leq 0.05$ versus control (CTL); #, $p \leq 0.05$ versus siCTL+ OGD/Rx 72h. **D-G.** Effects of siHDAC9 or scrambled siRNA on the expression of TfR1 (D), Fpn1 (E), Ft (F) and GPX4 (G) at 72h after OGD/Rx. Bars represent mean ± SD (n = 3); *, $p \leq 0.05$ versus control (CTL).

Suppl Fig 2. Effects of OGD/Rx on HDAC9, HIF-1 and Sp1 expression in primary cortical neurons.

A,C,D. Quantification of HDAC9 (A), HIF-1 (C) and Sp1 (D) mRNA at 48hr after OGD/Rx in primary cortical neurons treated with siHDAC9 or scrambled siRNA. **B.** Representative Western blot and quantification of HDAC9 protein levels at 48hr after OGD/Rx in cortical neurons treated with siHDAC9 or scrambled siRNA. Bars represent mean ± SD (n = 3); *, $p \leq 0.05$ versus normoxic control (CTL).

Suppl Fig 3. Efficacy of the silencing of HDAC9 and TfR1, and of GPX4 overexpression

A-B. Quantification of HDAC9 mRNA levels in bEND3 cells treated with siHDAC9 (A) and in mouse brain treated with siHDAC9 by icv route of administration (B). **C-D**. Quantification of TfR1 mRNA after siRNA transfection (C) and of GPX4 mRNA after plasmid transfection in primary cortical neurons. Bars represent mean ± SD (n = 3); *, $p \leq 0.05$ versus siCTL or empty vector.

Suppl Fig 4. Effects of siHDAC1, siHDAC2, siHDAC4 and siHDAC5 on OGD/Rx-induced TfR1 increase and GPX4 reduction in primary cortical neurons.

A-D. Representative Western blot and quantification of HDAC1 (A), HDAC2 (B), HDAC4 (C) and HDAC5 (D) in primary cortical neurons at 24hr and 48hr after OGD/Rx. **E-F.** Quantification of TfR1 (E) and GPX4 (F) mRNAs after siRNA transfection of siHDAC1, siHDAC2, siHDAC4 or siHDAC5 at OGD/Rx 48h. Bars represent mean ± SD (n = 3); *, $p \leq 0.05$ versus normoxic control (CTL).

Suppl Fig 5. HDAC9 immunosignal in temporoparietal cortex of mice subjected to tMCAO.

Confocal images of temporoparietal cortex of mice displaying HDAC9 (red), neuronal marker NeuN (green), and nuclear marker Hoechst (blue) signals in sham operated, tMCAO and tMCAO+siHDAC9 animals. Arrows indicates an increased HDAC9 immunosignal detected in the cytosolic compartment. Scale bars 25 µm.

Suppl Fig 6. Effect of siHDAC9 on the immunosignals of HIF-1, TfR1, Sp1, or GPX4 in temporoparietal cortex of mice subjected to tMCAO.
Confocal images of temporoparietal cortex of mice displaying HIF-1 (A, red), TfR1 (B, red), Sp1 (C, red), or GPX4 (D, red) immunosignals and their respective neuronal marker NeuN (green), and nuclear marker Hoechst (blue) signals in sham operated, tMCAO and tMCAO+siHDAC9 animals. Scale bars 25 µm.

Suppl Fig 7. Quantification of fluorescence intensity of HDAC9, HIF-1, TfR1, Sp1, or GPX4 in temporoparietal cortex of ischemic mice treated with siHDAC9 or siRNA scramble. Fluorescence intensity per photographic field (mm2) of HDAC9 labeling (A), HIF-1 labeling (B); TfR1 labeling (C), Sp1 labeling (D), GPX4 labeling (E) in perischemic temporopariental cortex of mice treated with siHDAC9 or siRNA scramble by icv injection and subjected to tMCAO 24 hours. Data are expressed as mean ± SEM (n = 3 for each group). *p < 0.05 versus sham-operated animals (Sham). P values were obtained using one-way ANOVA with Newman Keuls's correction for multiple comparisons.

Suppl Fig 8. HIF-1 and TfR1 immunosignal in temporoparietal cortex of ischemic mice.
Confocal images of temporoparietal cortex of mice displaying HIF-1(red), microglial marker Iba1 (green), and nuclear marker Hoechst (blue) signals in mice subjected to tMCAO 24hr. Arrows indicates the colocalization between HIF-1 and Iba1, and between TfR1 and Iba1. Scale bars 75 µm.

Suppl Fig 9. HIF-1 and TfR1 immunosignal in temporoparietal cortex of ischemic mice.
Confocal images of temporoparietal cortex of mice displaying HIF-1(red), astrocytic marker GFAP (green), and nuclear marker Hoechst (blue) signals in mice subjected to tMCAO 24hr. Arrows indicates the absence of colocalization between HIF1 and GFAP, and between TfR1 and GFAP. Scale bars 25 µm.

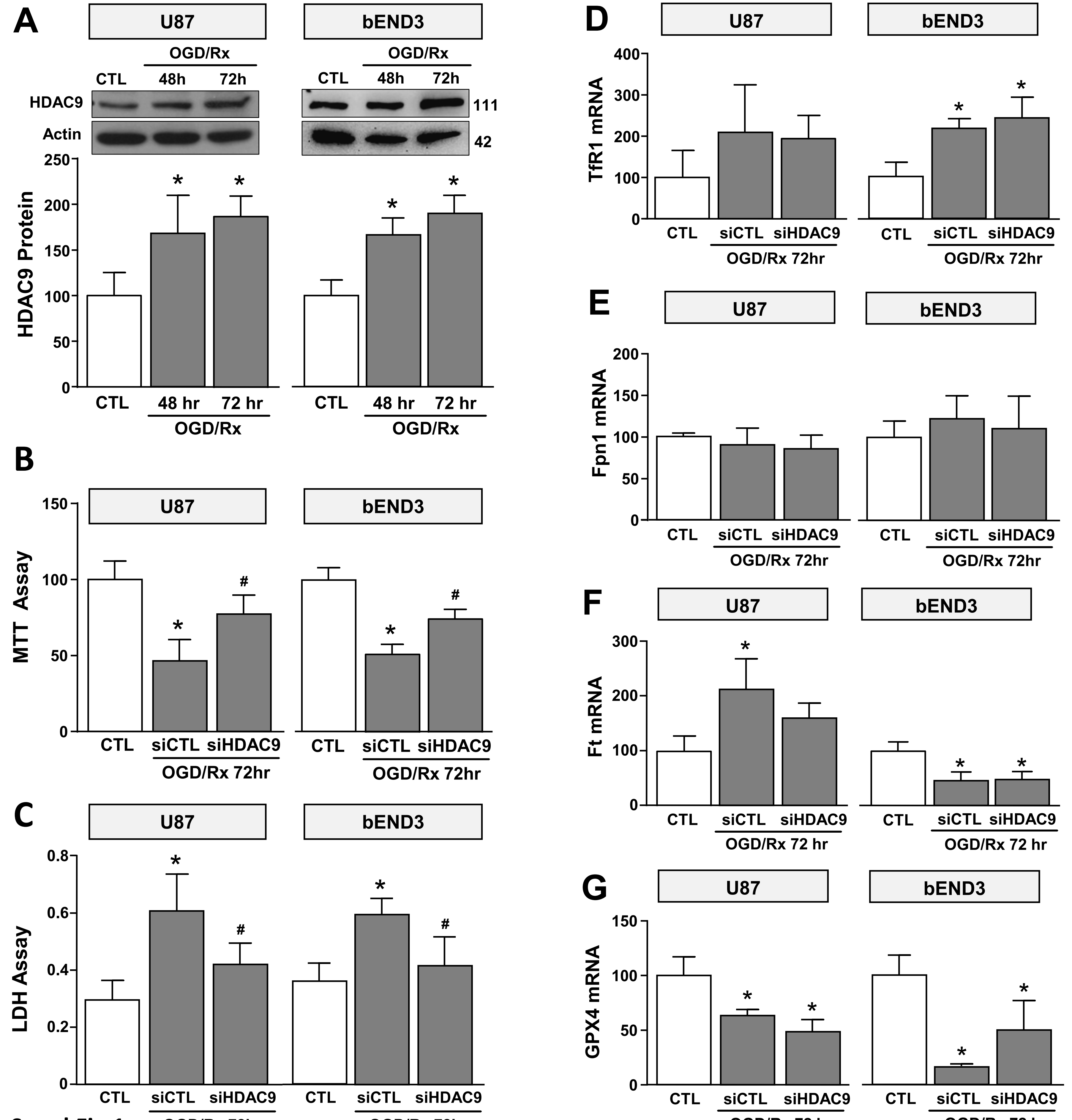
A
U87
bEND3
OGD/Rx
CTL
48h
72h
HDAC9
Actin
111
42
HDAC9 Protein
250
200
150
100
50
0
CTL
48 hr
72 hr
OGD/Rx
B
MTT Assay
150
100
50
0
CTL
siCTL
siHDAC9
OGD/Rx 72hr
C
LDH Assay
0.8
0.6
0.4
0.2
0
D
TfR1 mRNA
400
300
200
100
0
E
Fpn1 mRNA
F
Ft mRNA
OGD/Rx 72 hr
G
GPX4 mRNA
*
#

Suppl Fig.1

Cortical Neurons

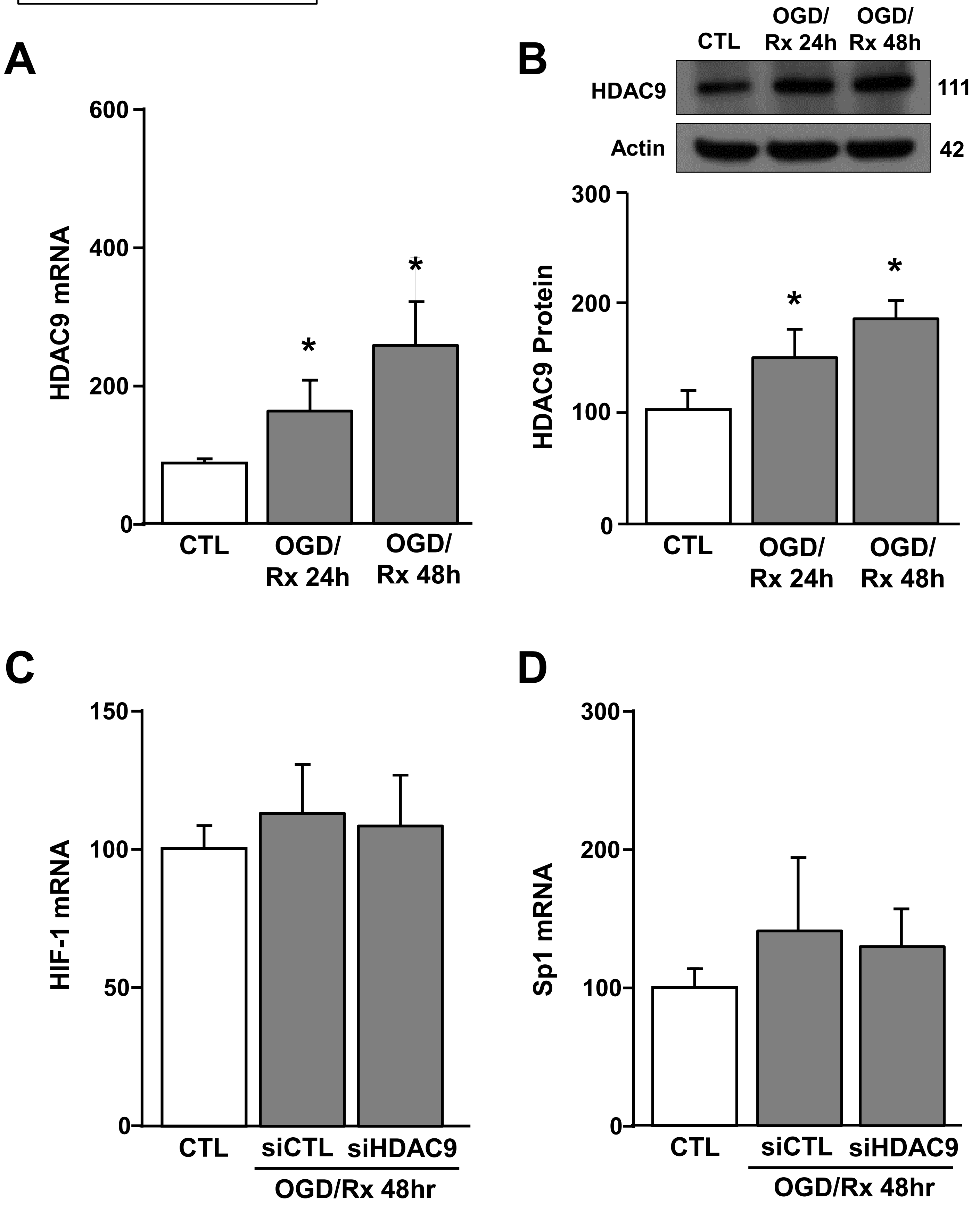

Suppl Fig.2

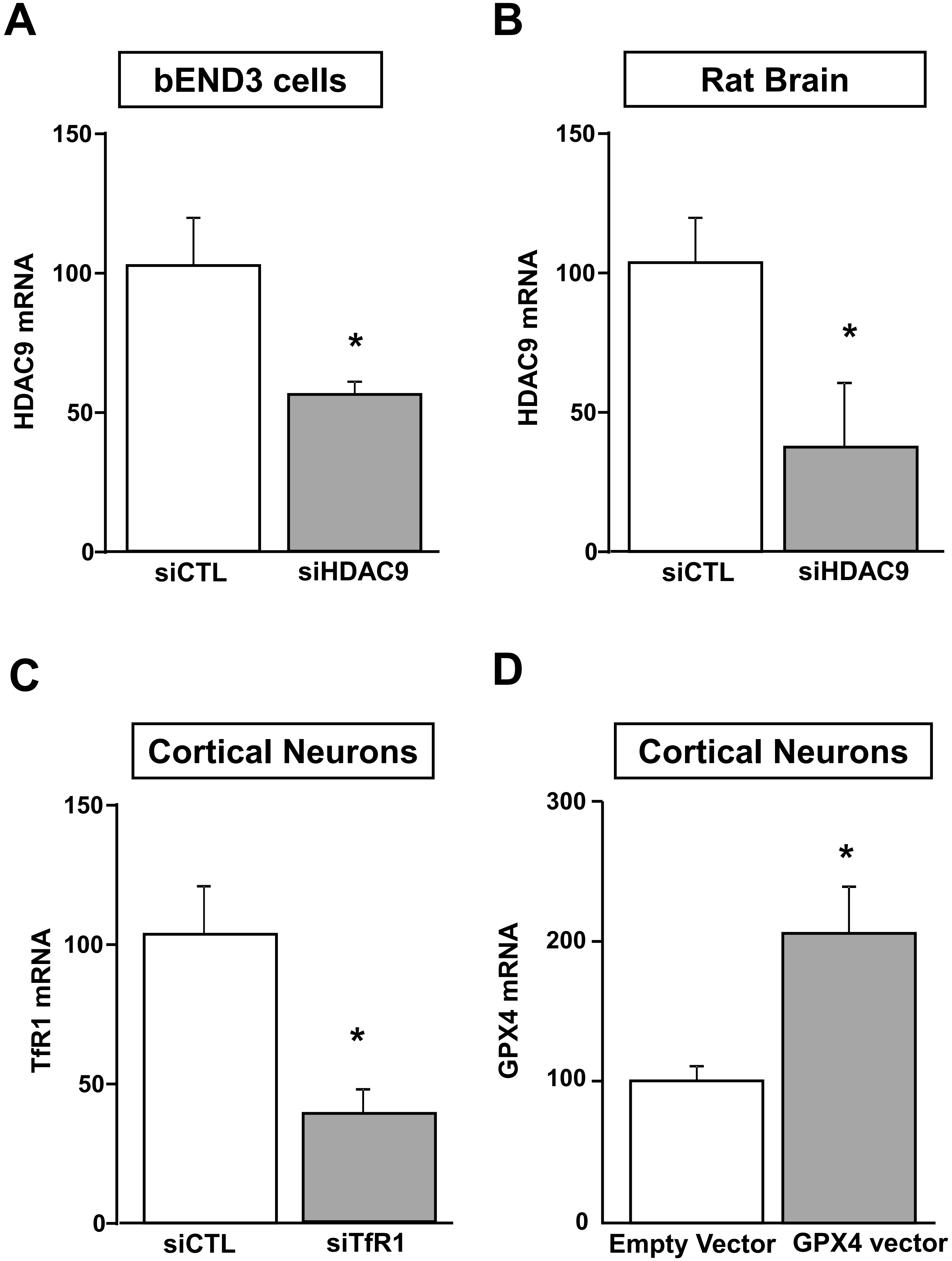
A
bEND3 cells
HDAC9 mRNA
150
100
50
0
*
siCTL
siHDAC9
B
Rat Brain
HDAC9 mRNA
150
100
50
0
*
siCTL
siHDAC9
C
Cortical Neurons
TfR1 mRNA
150
100
50
0
*
siCTL
siTfR1
D
Cortical Neurons
GPX4 mRNA
300
200
100
0
*
Empty Vector
GPX4 vector

Suppl Fig.3

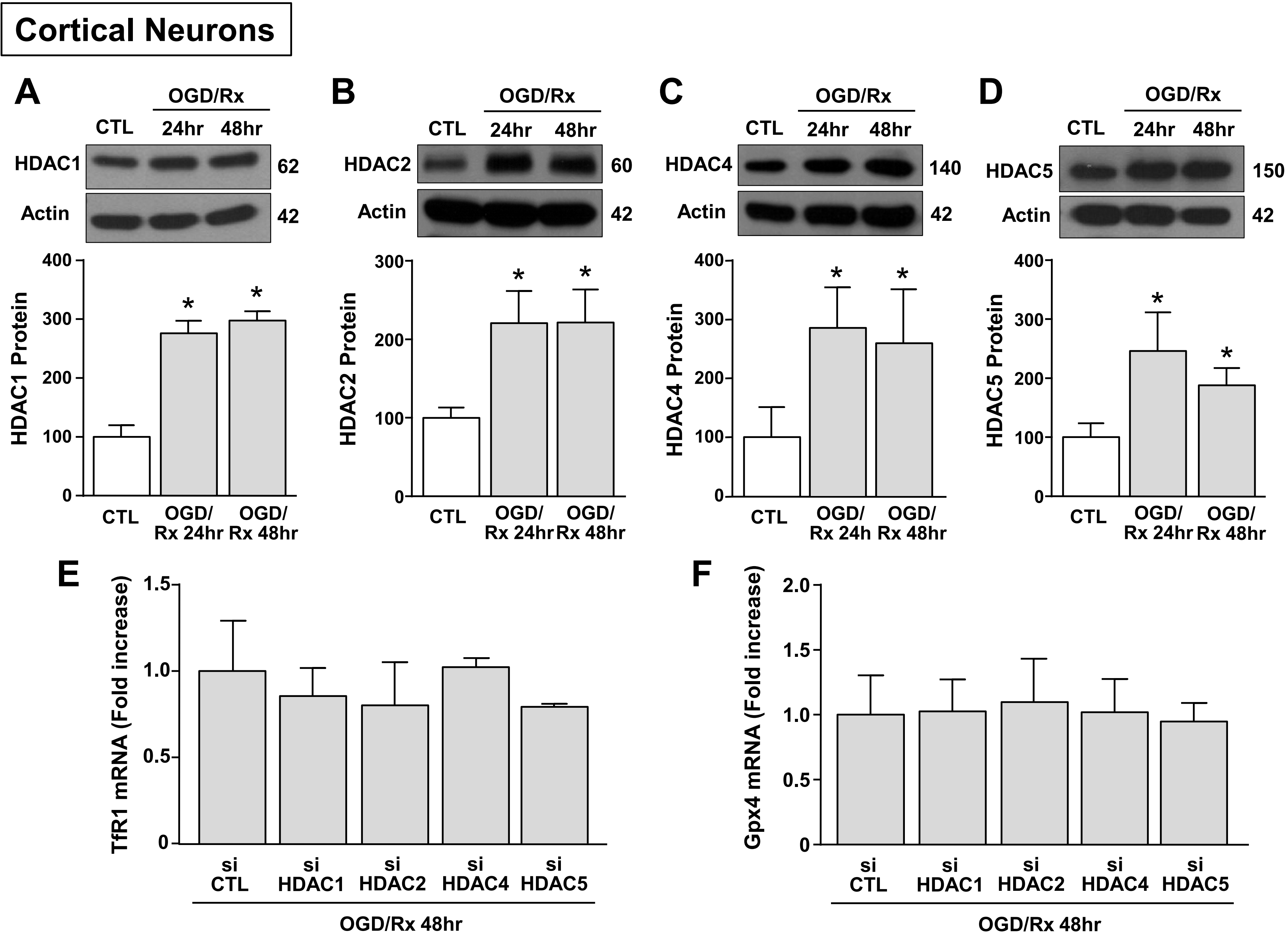
Cortical Neurons
A
OGD/Rx
CTL
24hr
48hr
HDAC1
62
Actin
42
HDAC1 Protein
CTL
OGD/Rx 24hr
OGD/Rx 48hr
B
HDAC2
60
HDAC2 Protein
C
HDAC4
140
HDAC4 Protein
OGD/Rx 24h
D
HDAC5
150
HDAC5 Protein
E
TfR1 mRNA (Fold increase)
si CTL
si HDAC1
si HDAC2
si HDAC4
si HDAC5
OGD/Rx 48hr
F
Gpx4 mRNA (Fold increase)

Suppl. Fig. 4

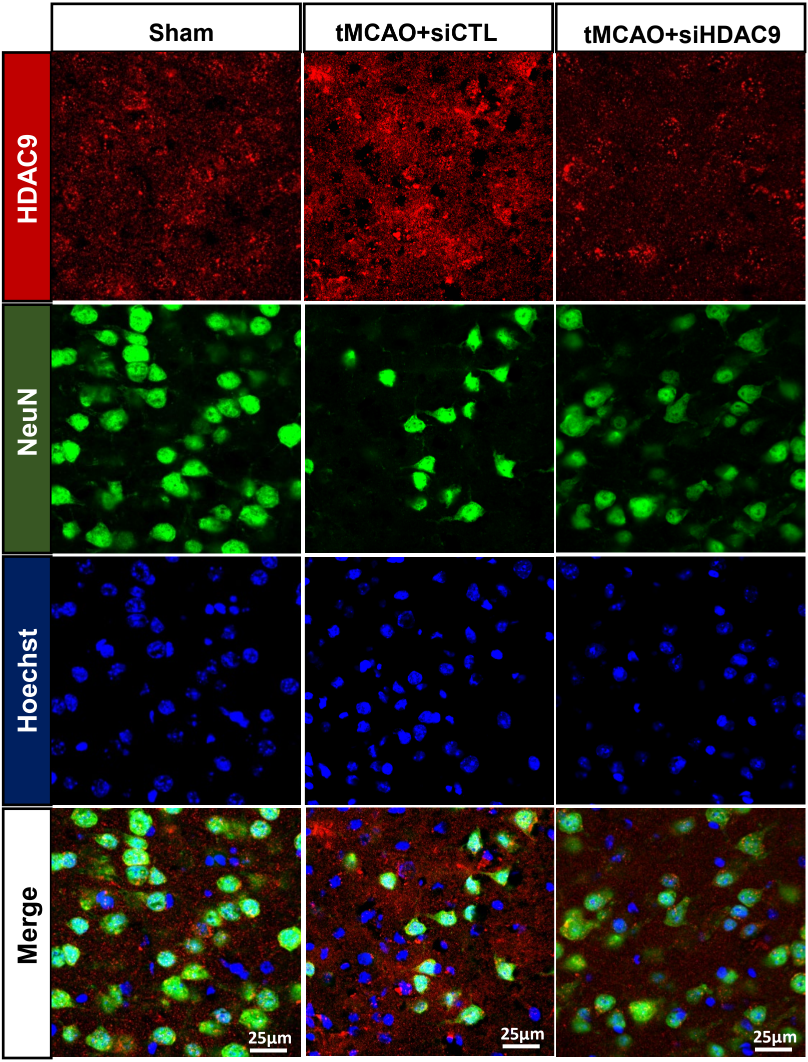

Sham
tMCAO+siCTL
tMCAO+siHDAC9
HDAC9
NeuN
Hoechst
Merge
25µm
25µm
25µm

Suppl. Fig. 5

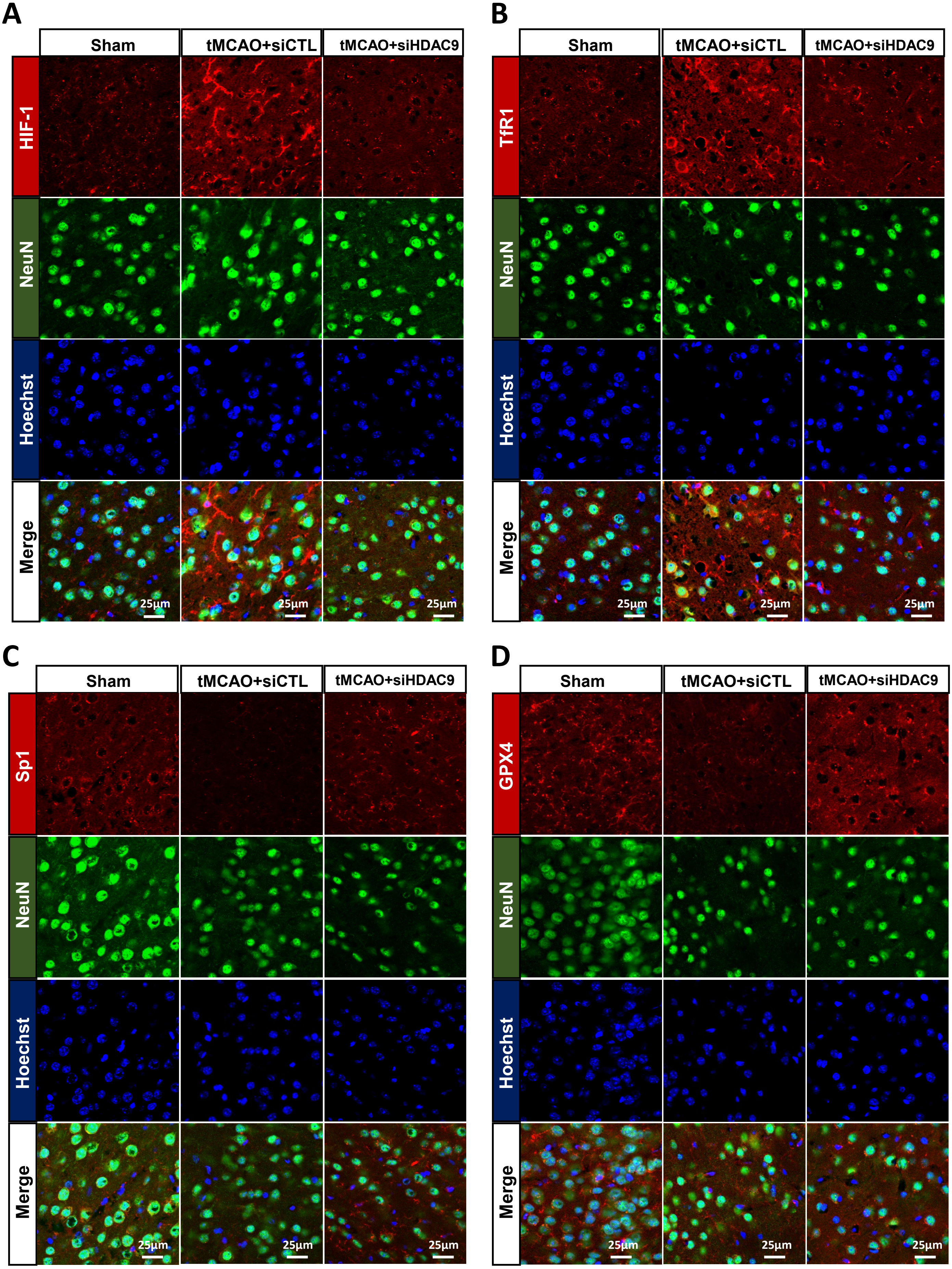

A
Sham
tMCAO+siCTL
tMCAO+siHDAC9
HIF-1
NeuN
Hoechst
Merge
25µm
B
Sham
tMCAO+siCTL
tMCAO+siHDAC9
TfR1
NeuN
Hoechst
Merge
25µm
C
Sham
tMCAO+siCTL
tMCAO+siHDAC9
Sp1
NeuN
Hoechst
Merge
25µm
D
Sham
tMCAO+siCTL
tMCAO+siHDAC9
GPX4
NeuN
Hoechst
Merge
25µm

Suppl. Fig. 6

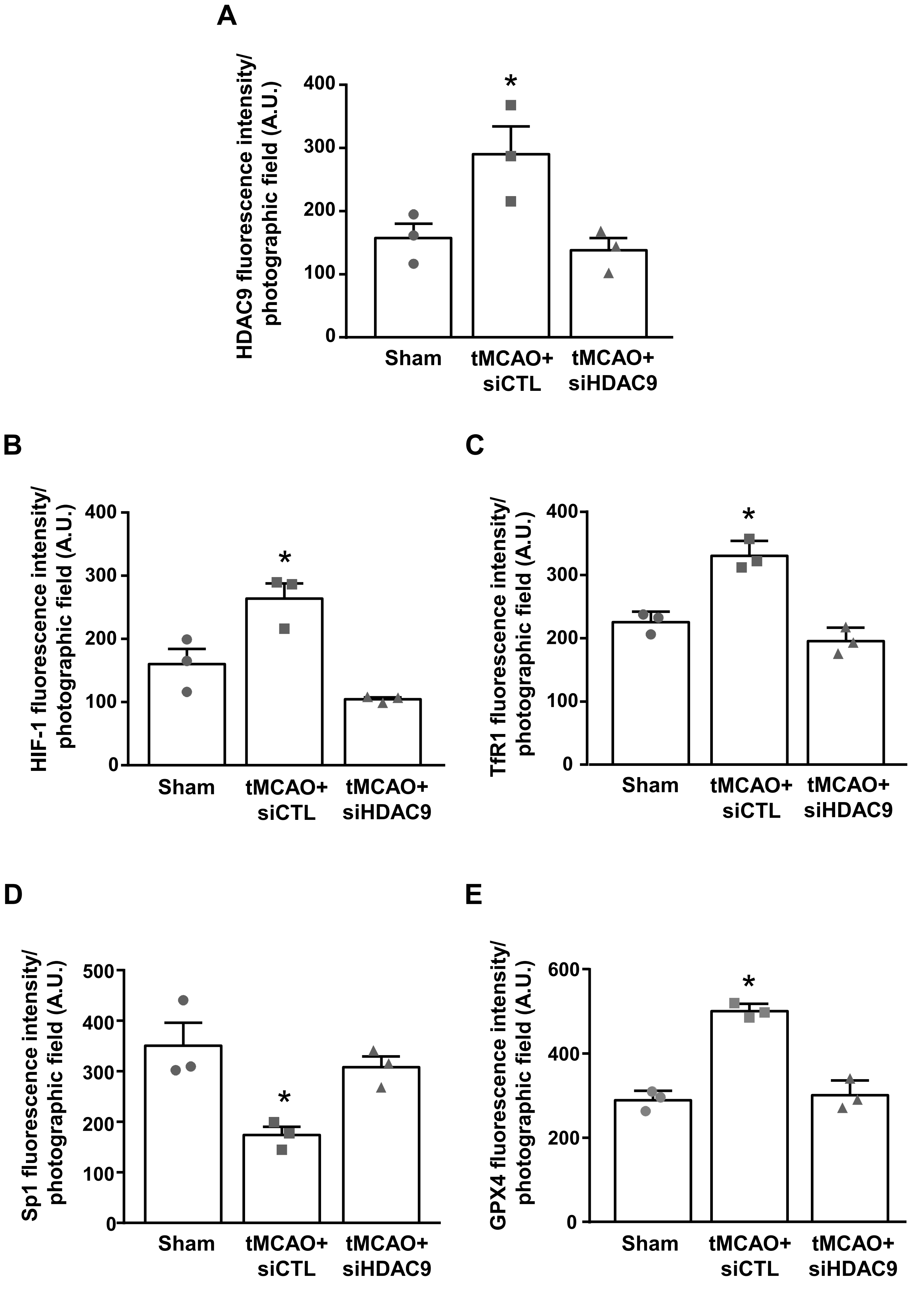
A
HDAC9 fluorescence intensity/
photographic field (A.U.)
400
300
200
100
0
*
Sham
tMCAO+
siCTL
tMCAO+
siHDAC9
B
HIF-1 fluorescence intensity/
photographic field (A.U.)
400
300
200
100
0
*
Sham
tMCAO+
siCTL
tMCAO+
siHDAC9
C
TfR1 fluorescence intensity/
photographic field (A.U.)
400
300
200
100
0
*
Sham
tMCAO+
siCTL
tMCAO+
siHDAC9
D
Sp1 fluorescence intensity/
photographic field (A.U.)
500
400
300
200
100
0
*
Sham
tMCAO+
siCTL
tMCAO+
siHDAC9
E
GPX4 fluorescence intensity/
photographic field (A.U.)
600
400
200
0
*
Sham
tMCAO+
siCTL
tMCAO+
siHDAC9

Suppl. Fig. 7

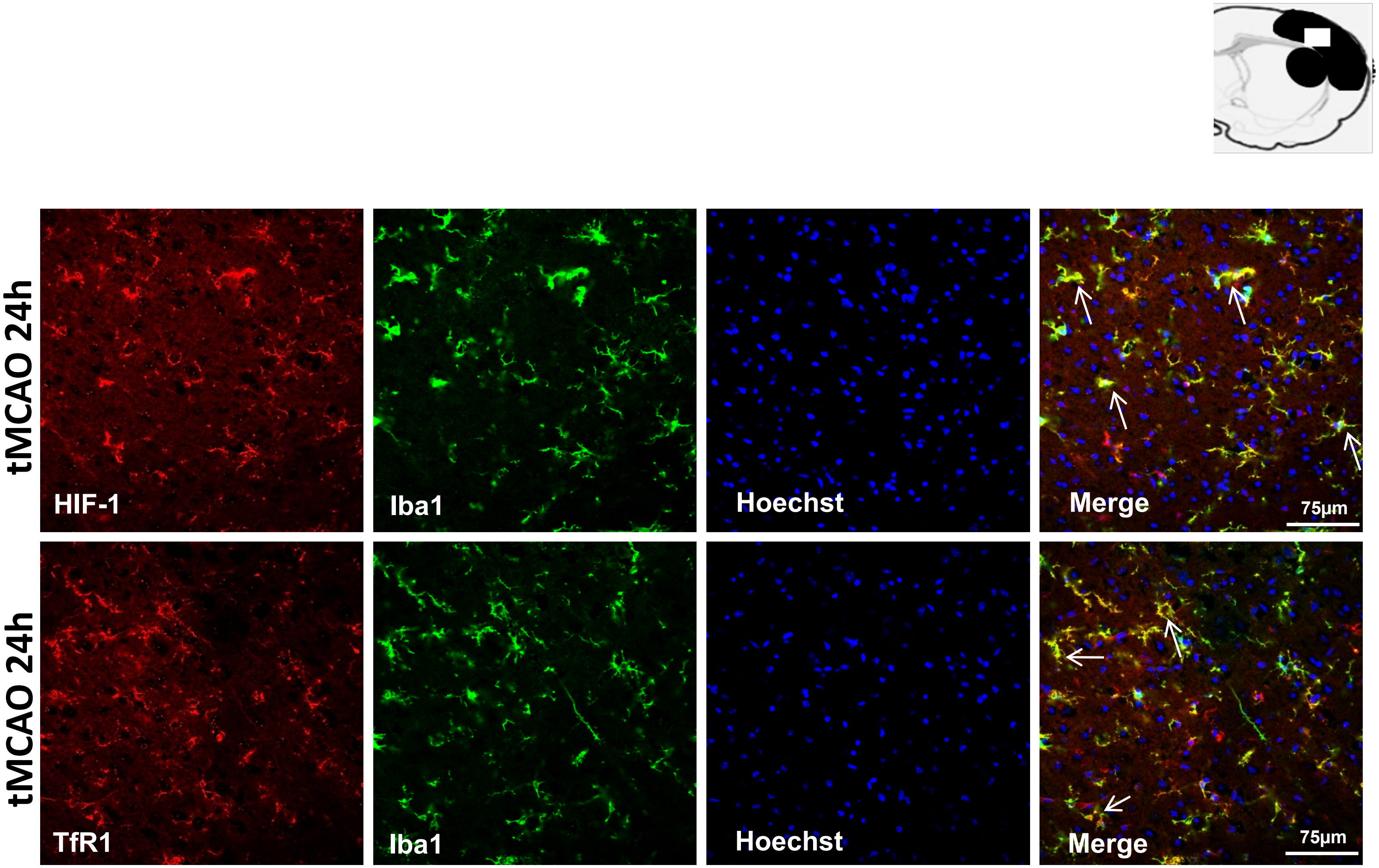
tMCAO 24h
HIF-1
Iba1
Hoechst
Merge
75µm
tMCAO 24h
TfR1
Iba1
Hoechst
Merge
75µm

Suppl. Fig. 8

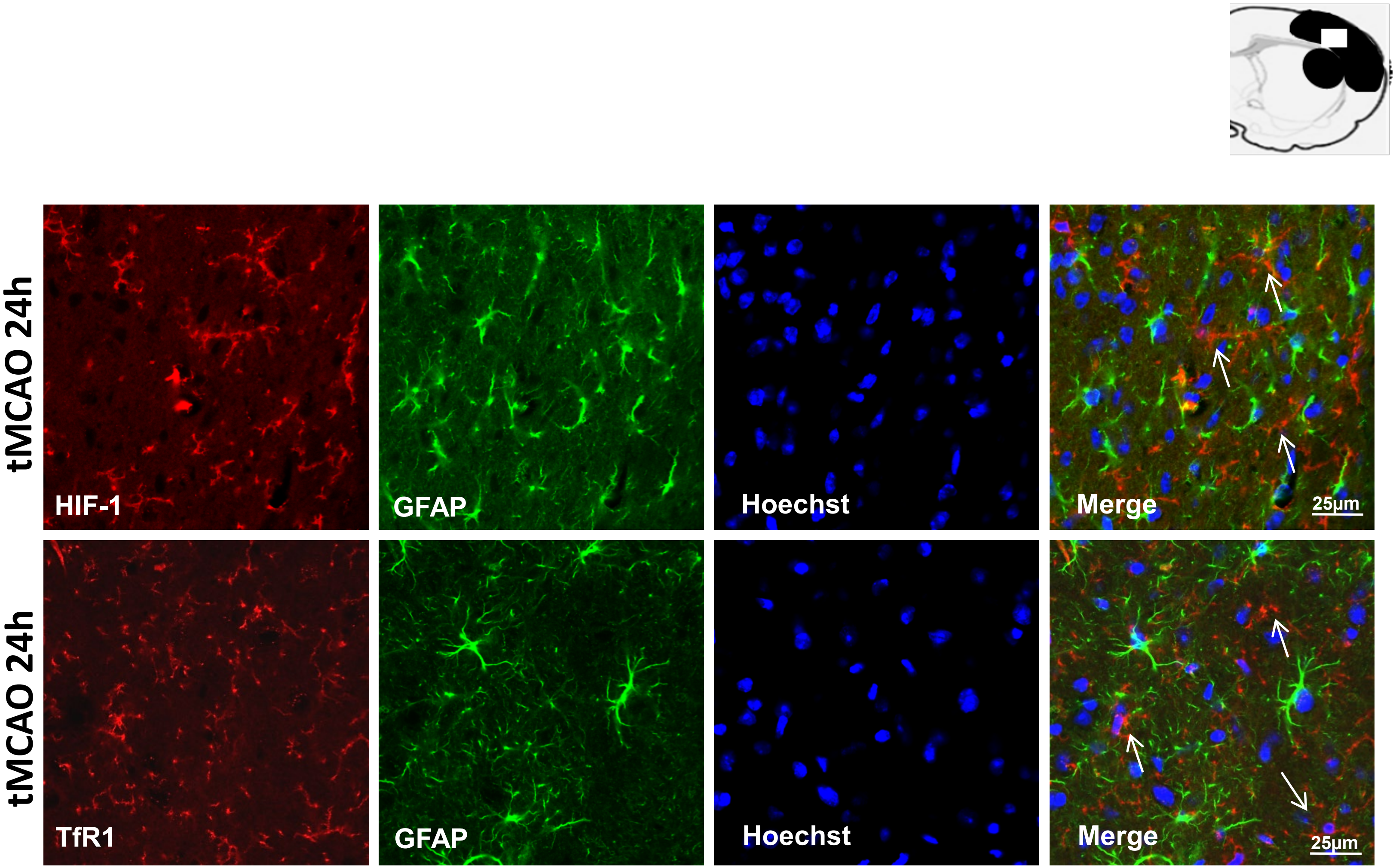
tMCAO 24h
HIF-1
GFAP
Hoechst
Merge
25μm
tMCAO 24h
TfR1
GFAP
Hoechst
Merge
25μm

Suppl. Fig. 9
